# The Summer 2019–2020 Wildfires in East Coast Australia and Their Impacts on Air Quality and Health in New South Wales, Australia

**DOI:** 10.3390/ijerph18073538

**Published:** 2021-03-29

**Authors:** Hiep Duc Nguyen, Merched Azzi, Stephen White, David Salter, Toan Trieu, Geoffrey Morgan, Mahmudur Rahman, Sean Watt, Matthew Riley, Lisa Tzu-Chi Chang, Xavier Barthelemy, David Fuchs, Kaitlyn Lieschke, Huynh Nguyen

**Affiliations:** 1 Department of Planning, Industry and Environment, P.O. Box 29, Lidcombe, NSW 2141, Australia; merched.azzi@environment.nsw.gov.au (M.A.); Stephen.White@environment.nsw.gov.au (S.W.); david.salter@environment.nsw.gov.au (D.S.); toan.trieu@environment.nsw.gov.au (T.T.); Md-Mahmudur.Rahman@environment.nsw.gov.au (M.R.); Sean.Watt@environment.nsw.gov.au (S.W.); matthew.riley@environment.nsw.gov.au (M.R.); LisaTzu-Chi.Chang@environment.nsw.gov.au (L.T.-C.C.); xavier.barthelemy@environment.nsw.gov.au (X.B.); david.fuchs@environment.nsw.gov.au (D.F.); kaitlyn.lieschke@environment.nsw.gov.au (K.L.); 2 Environmental Quality, Atmospheric Science and Climate Change Research Group, Ton Duc Thang University, Ho Chi Minh City 700000, Vietnam; 3 Faculty of Environment and Labor Safety, Ton Duc Thang University, Ho Chi Minh City 700000, Vietnam; 4 University Centre of Rural Health, North Coast, University of Sydney, Lismore, NSW 2480, Australia; geoffrey.morgan@sydney.edu.au; 5 Faculty of Engineering & Information Technology, University of Technology Sydney, Ultimo, NSW 2007, Australia; HUYNHANHDUY.NGUYEN@student.uts.edu.au

**Keywords:** wildfires, South East Australia, WRF-Chem, pollutant transport, air quality effect, health impact

## Abstract

The 2019–2020 summer wildfire event on the east coast of Australia was a series of major wildfires occurring from November 2019 to end of January 2020 across the states of Queensland, New South Wales (NSW), Victoria and South Australia. The wildfires were unprecedent in scope and the extensive character of the wildfires caused smoke pollutants to be transported not only to New Zealand, but also across the Pacific Ocean to South America. At the peak of the wildfires, smoke plumes were injected into the stratosphere at a height of up to 25 km and hence transported across the globe. The meteorological and air quality Weather Research and Forecasting with Chemistry (WRF-Chem) model is used together with the air quality monitoring data collected during the bushfire period and remote sensing data from the Moderate Resolution Imaging Spectroradiometer (MODIS) and Cloud-Aerosol Lidar and Infrared Pathfinder Satellite Observation (CALIPSO) satellites to determine the extent of the wildfires, the pollutant transport and their impacts on air quality and health of the exposed population in NSW. The results showed that the WRF-Chem model using Fire Emission Inventory (FINN) from National Center for Atmospheric Research (NCAR) to simulate the dispersion and transport of pollutants from wildfires predicted the daily concentration of PM_2.5_ having the correlation (R^2^) and index of agreement (IOA) from 0.6 to 0.75 and 0.61 to 0.86, respectively, when compared with the ground-based data. The impact on health endpoints such as mortality and respiratory and cardiovascular diseases hospitalizations across the modelling domain was then estimated. The estimated health impact on each of the Australian Bureau of Statistics (ABS) census districts (SA4) of New South Wales was calculated based on epidemiological assumptions of the impact function and incidence rate data from the 2016 ABS and NSW Department of Health statistical health records. Summing up all SA4 census district results over NSW, we estimated that there were 247 (CI: 89, 409) premature deaths, 437 (CI: 81, 984) cardiovascular diseases hospitalizations and 1535 (CI: 493, 2087) respiratory diseases hospitalizations in NSW over the period from 1 November 2019 to 8 January 2020. The results are comparable with a previous study based only on observation data, but the results in this study provide much more spatially and temporally detailed data with regard to the health impact from the summer 2019–2020 wildfires.

## 1. Introduction

As a dry continent, Australia has been prone to having frequent wildfires as part of the landscape ecology at least since the Middle Eocene (~40 million years ago) [1]. Some flora species have adapted to wildfire conditions, such as *Banksia*, *B. serrata* on the east coast and *B. candolleana* in south-western Australia, to be able to survive them [2]. These species also need fires to regenerate. As natives to the land, the Aboriginals were the first to practice wildfire management to reduce the impact of wildfires on the environment and communities. Since the European settlement in the 18th century, agriculture and urban growth have changed the landscape, while wildfires continue and affect the communities more often [3]. Currently, hazardous reduction burnings (HRBs) to reduce fuel load during late boreal autumn and winter amount to the main tool to manage wildfires which occur frequently in late spring and summer, especially in El-Niño years when dry and less rainy conditions occur over most of Australia [3,4]. 

**Figure 1:**
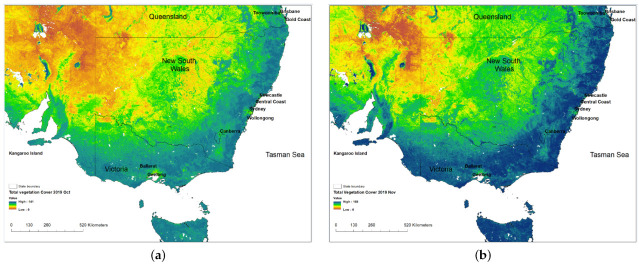
Vegetation cover over southeastern Australia in October (**a**) and November 2019 (**b**) (source: Australia monthly cover. Available online: http://www-data.wron.csiro.au/remotesensing/MODIS/products/public/v310/australia/monthly/cover/, accessed on 27 March 2021). In November, the rain alleviated the drought condition in midwestern NSW and promoted the green growth with increased vegetation cover along the coast of NSW and Victoria.

The state of New South Wales (NSW) and most of eastern Australia was in drought from 2018 to the end of 2019. In October 2019, spring rain then relieved part of western NSW (Figure 1b). This resulted in increasing vegetation growth which increased the likelihood of fires during the subsequent summer due to more fuel load available and when meteorological conditions were more favorable to ignite fires. As shown in Figure 1, the change in vegetation cover from October (Figure 1a) when dry bare land covered much of western NSW to November (Figure 1b) after spring rain was significant. After the rain, the drought was then only confined to far western NSW and higher vegetation growth occurred throughout much of the rest of NSW and Victoria.

The soil moisture map for the months of November, December 2019 and January 2020 (as shown in Figure S1 in the Supplementary Materials) revealed that the upper soil and root zone soil moisture as a fraction of fullness was low in most of NSW, northwestern Victoria and South Australia, especially in December 2019. Wildfires in the northern NSW and central Queensland occurred earlier in austral spring from 7 September to the end of the month at a small scale. However, more favorable conditions, such as high temperature, low humidity, low soil moisture taking the Forest Fire Danger Index (FFDI) to a dangerous level, occurred in the summer 2019 in eastern Australia. 

With low soil moisture and high fuel load along the coast, the occurrence of high temperature and low humidity in late spring and early summer of 2019 caused wildfires to start and the fires gradually consumed much of the southeastern coastal areas of Australia where relatively high fuel load was concentrated. This convergence of extreme events or conditions (low soil moisture, high fuel load, high temperature and low humidity) over the large region of the southeastern coast of Australia resulted in a compound megafire event consisting of many wildfires lasting from early November 2019 to mid-January 2020. This compound extreme climate event can be expected to occur more frequently in the future under climate change scenarios of a global warming due to increasing anthropogenic greenhouse emissions.

For NSW, the late austral spring and summer wildfires started in the northern part of the state and the Blue Mountains near Sydney in late October, November, December 2019 followed by early January 2020 and onwards in the southern part of the state to the border with Victoria, with large areas near the coast on fire. Victoria and South Australia also had many fires during January starting from 3–5 January 2020. The progress of the wildfires can be seen from remote sensing data (Figure S2 in the Supplementary Materials) which show the monthly vegetation cover loss from November to December 2019 due to wildfires (vegetation cover in December subtracted from November 2019) and from December 2019 to January 2020 over Australia and over NSW. The progress of the summer 2019–2020 wildfires can be divided into two stages: north coast of NSW, Blue Mountains in November and December 2019 and the south coast toward Victoria, Victorian East Gippsland, the Mallee region west of Bendigo, South Australia and Kangaroo Island and southwestern Australia in January 2020. The summer 2019–2020 wildfires were unprecedented in scale as the fires occurred in several states and the burning area in NSW alone was 5.68 million ha, in Victoria—1.58 million ha [5]. Thirty-three people lost their lives due to this summer 2019–2020 wildfires [6].

**Figure 2:**
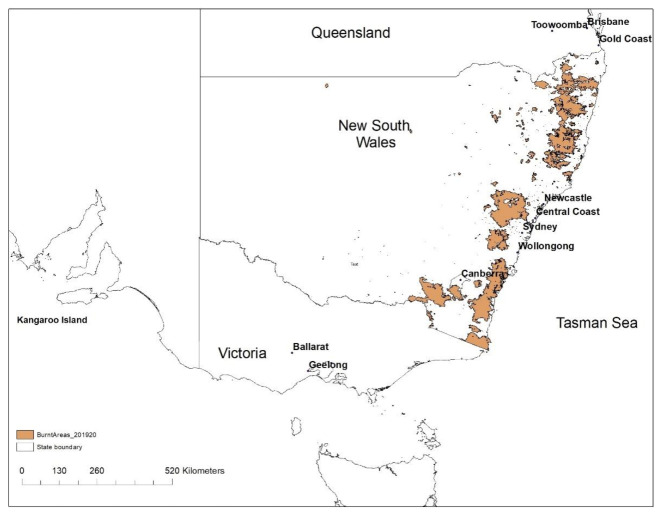
Total burned areas in NSW in the summer 2019–2020 bushfire event (Source: Department of Planning, Industry and Environment, DPIE).

The 2019–2020 summer wildfires were so intense and widespread that it is now known as the Black Summer wildfires. Figure 2 shows the total area burned for NSW after the summer 2019–2020 bushfire event. The intensity of the wildfires caused smoke plumes and emitted air pollutants to be dispersed over a wide region in the east coast of Australia and across the Tasman Sea to New Zealand in January 2020. Plumes of smoke and pollutants injected high into the atmosphere, into the upper troposphere and at times into the stratosphere caused smoke particles to be transported across the Pacific to South America (Argentina and Chile) and beyond [7]. The effect of the wildfires on the environment and population exposure to emitted air pollutants were studied by many authors [8,9,10,11,12,13,14]. Wildfire-emitted pollutants deteriorate ambient air quality affecting people’s health. Deposition of fire pollutants, such as Fe, on water body surfaces like the ocean also stimulates the growth of phytoplankton. After dust deposition, pyrogenic (fires and anthropogenic combustion) Fe is the second largest source of Fe deposition on the surface of the ocean and is a significant contributor to daily, seasonal and interannual variability of Fe in the ocean basins [15]. 

It was estimated the wildfires released 400 megatons of carbon dioxide (CO_2_) into the atmosphere, equal to three-quarters of Australian industry emissions in 2018–2019 [16]. Besides greenhouse gas CO_2_, wildfires emitted a wide range of pollutants such as nitrogen oxides (NO_x_), particles (PM_10_, PM_2.5_), various species of hydrocarbons. These pollutants can cause serious health effects in the exposed population, especially in the susceptible people such as children and the elderly. In 2018, Knibbs et al. [17] showed exposure of NO_2_ in children results in reduced lung function and in asthmatic conditions in susceptible children. Increase in PM_2.5_ concentration was shown to have an impact on a number of health endpoints of the exposed population, such as the increase in mortality rate and respiratory and cardiovascular diseases hospitalizations [8]. Health costs of population exposure to smoke particles from wildfire events in Tasmania between 2010 and 2019 were estimated to be AUS$ 16 million annually on average, but for extreme wildfires, this figure increased up to AUS$ 34 million [18]. 

A number of studies has been recently conducted to estimate the impact of the 2019–2020 wildfires [9,19]. A review of the effect of wildfires on a number of health endpoints including respiratory, chronic obstructive pulmonary disease (COPD), cardiovascular and asthma hospital admissions was also published [8]. As the summer 2019–2020 wildfires were unprecedented in temporal duration and spatial scale and affecting most of the populated east coast of Australia covering three states, Queensland, New South Wales and Victoria, and air quality monitoring data is sparse across this large domain and not adequate to determine the exposure accurately for health impact, this study used a modelling approach to calculate the ground pollutant concentration at high spatial resolution for air quality and health impact investigation. 

Previous studies used air quality models such as Conformal Cubic Atmospheric Model- Chemical Transport Model (CCAM–CTM) [10] and Weather Research and Forecasting-Community Multiscale Air Quality Modeling System (WRF-CMAQ) [20,21] to assess the impact of fires on air quality by determining the dispersion and transport of emitted pollutants across the study domain. In our study, to determine the impact of wildfires on air quality and health due to population exposure to particulate pollutants, the WRF-Chem air quality model was used to predict the increase in PM_2.5_ concentration over NSW due to wildfires during the summer 2019–2020 wildfires. The increase in PM_2.5_ at each grid point in the modelling domain due to wildfires was used to assess the impact on health endpoints. The results from the model were also evaluated using observed meteorological and air quality data from the NSW DPIE monitoring data network and remote sensing data from the MODIS Aqua/Terra and CALIPSO lidar satellites.

## 2. Materials and Methods

**Figure 3:**
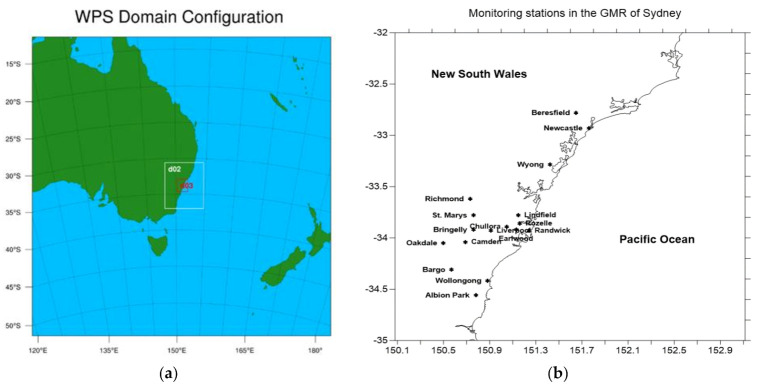
(**a**) WRF-Chem domain configuration over eastern Australia; (**b**) DPIE air quality monitoring stations in the Greater Metropolitan Region (GMR) of Sydney which is defined as domain d03 (the red rectangle in (**a**)).

As the focus of the study was on fine particle concentrations (PM_2.5_) due to wildfires and their impact on health, air quality monitoring data, especially PM_2.5_, for the wildfires period were obtained from the Department of Industry, Planning and Environment (DPIE) of New South Wales’s air quality monitoring network. The monitoring data were used to assess the impact of wildfires on air quality and to validate the model prediction. Figure 3b shows the monitoring stations in NSW. During the wildfires period, additional monitoring stations, such as Port Macquarie station, were deployed in the northern NSW. 

In addition to the ground-based monitoring data, the satellite data were used for comparison with the model prediction and to determine the extent of pollutant transport. Satellite data of hot spots, smoke plumes and aerosol optical depth (AOD) were obtained from the MODIS Aqua/Terra satellites while aerosol vertical profiles came from the Cloud-Aerosol Lidar with Orthogonal Polarization (CALIOP) lidar onboard the CALIPSO satellite. These data provided daily spatial distribution of aerosols and smoke plumes over the east coast of Australia from Victoria to New South Wales and Queensland while vertical aerosol profiles from the CALIOP lidar provided the aerosol structure above the ground at one- or two-day intervals over the bushfire regions. Output from the MERRA-2 (Modern-Era Retrospective Analysis for Research and Applications, Version 2) reanalysis system developed by NASA was used to determine the concentration distribution of PM_2.5_. MERRA-2 is an air quality model with data assimilation from various observation sources, including satellites. 

For higher resolution output, we used WRF-Chem with emissions from the Fire Emission Inventory from NCAR (FINN) to model the air quality over eastern Australia. The FINN is based on the hot spots (thermal anomalies) detected by MODIS Terra/Aqua satellites and on the vegetation type assigned to each fire pixel based on the MODIS Collection 5 Land Cover Type (LCT) product based on 16 land cover/land use (LULC) classes from the IGBP (International Geosphere–Biosphere Program) land cover classification. Fuel load density at each active fire location was determined using the MODIS Vegetation Continuous Fields (VCF) product. The emission factors of emitted species from fires for each of the vegetation types were obtained from previously published studies, such as from [22] for savanna and grassland and crop residue types. The maximum burned area at each fire pixel was assumed to be 1 km^2^ but scaled to the VCF fuel cover data if present for that pixel. Finally, the fraction of the biomass assumed to burn at each fire point was assigned as a function of tree cover [23]. From the information above, the emissions from fire pixels was then calculated. The FINN provides emissions of species suitable for air quality models [23]. The datasets are provided on the daily basis with 1-h time resolution and approximately 1 km^2^ spatial resolution globally for three different air chemistry mechanisms: GEOS-CHEM (Goddard Earth Observing System-Atmospheric Chemistry), MOZART-4 (Model for OZone and Related chemical Tracers 4) and SAPRC99 (State-wide Air Pollution Research Center, Version 1999). The datasets can be downloaded from the NCAR Atmospheric Chemistry Observations and Modeling website. In this study, the MOZART/GOCART (Goddard Chemistry Aerosol Radiation and Transport) option for chemistry was chosen for the WRF-Chem simulation. 

As for meteorological drivers used in WRF-Chem, the National Center for Environmental Prediction (NCEP) final analysis (FNL) reanalysis data provided the boundary and initial meteorological conditions for WRF-Chem to downscale to the modelling domain. In this study, the modelling domain was based on the Lambert projection with 386 × 386 grid points at the resolution of 12 km × 12 km. There were 32 model vertical sigma levels. The central reference coordinate was at 150.994° longitude and −33.921° latitude. 

Figure 3 shows the WRF-Chem simulation domain and the air quality monitoring stations in the GMR of Sydney. The data from these stations were used to validate the prediction from the model as well as determine the effect of wildfires on air quality. Table S1 in the Supplementary Materials shows some of the WRF-Chem physical and chemical configuration options as defined in WRF-Chem to be used in this study. 

Validation of the model simulation was performed by comparing the meteorological prediction and air quality prediction with observations at many monitoring sites in the DPIE NSW’s air quality monitoring network (as shown in Figure 3b) and the remote sensing data from satellites.

The simulation of air quality over eastern Australia allowed us to determine the ground concentration of PM_2.5_ due to wildfires across NSW. In the simulation, biogenic emissions were included but anthropogenic emission sources were turned off, so that only the effect of wildfires could be determined. Thus, the exposure and health impact on the population could be estimated. Health impact for different health endpoints (mortality, hospitalizations for cardiovascular and respiratory diseases) is based on the impact function (IF) calculated as follows:

IF = RR^ΔX/10^ (1)

where RR is the relative risk of a health endpoint from an increase of 10 μg/m^3^ PM_2.5_ pollutant concentration and is determined from epidemiological studies. After that, the attributable number (AN) of the impact on a health endpoint due to an increase of ΔX concentration in a population (Pop) with a known incidence rate of the health endpoint is calculated as follows:

AN = (IF − 1) × Pop × incidence rate.(2)

We focused on the simulation period from 1 November 2019 to 8 January 2020 when the wildfires mostly concentrated in New South Wales (NSW), Australian Capital Territory (ACT) and Victoria (VIC) where the majority of population in southeastern Australia live. The 2016 population data and the mortality rate for each of the census district (SA4 level) were obtained from the Australian Bureau of Statistics (ABS). As the ABS does not provide hospitalization data, the incidence rates for cardiac diseases hospitalizations were obtained from the NSW Department of Health for each of the local government areas (LGAs), for respiratory diseases hospitalizations—for each of the local health districts (LHDs). The average of the incidence rates in three years (2016, 2015 and 2014) for cardiac and respiratory diseases hospitalizations were used in this study. The predicted daily PM_2.5_ concentration grids were superimposed on the SA4s shapefiles to obtain the average PM_2.5_ concentration in each of the SA4s. Incidence rates for cardiac and respiratory diseases hospitalizations were projected to each of the SA4s from the LGA and LHD shapefiles and calculated as the average of the rates of intersected LGAs or LHDs with the SA4. The attributable number (AN) was calculated for each of the health endpoints based on the incidence rates in SA4s. These ANs were then summed over all of the SA4s in NSW to estimate the effect of increased PM_2.5_ from wildfires on population health. 

The RR values for premature mortality and cardiovascular diseases hospitalization health endpoints used in this study were based on the World Health Organization’s (WHO) Health Risks of Air Pollution in Europe project (HRAPIE) recommendations (2013) [24] regarding short-term concentration response coefficients for mortality and CVD diseases.

RR (mortality) = 1.0123 (CI: 1.0045, 1.0201)

RR (CVD) = 1.0091 (CI: 1.0017, 1.0201)

where CI is the confidence interval. 

The RR value (1.23%) for mortality above corresponds to those in Xu et al. (2020) study. In their study, they reviewed the effect of wildfire events in literature and reported that the daily increase of 10 μg/m^3^ of the PM_2.5_ level is associated with an increase of 0.8–2.4% in the risk of death from any cause or nonaccidental death for up to 4 days after the exposure 

For respiratory hospitalizations, the RR of PM_2.5_ of 1.03 (CI: 1.01, 1.04) based on a study of the 2003 California wildfires [11] was used in this study. This value was also used in [10,12] for wildfires or biomass hazardous reduction burnings (HRBs) in Australia. 

Of the three RRs above (mortality, cardiovascular and respiratory diseases hospitalizations), the RR value for CVD is the smallest and reflects that the effect of PM_2.5_ on CVD hospitalizations is smaller than on mortality and respiratory diseases hospitalizations.

## 3. Results

### 3.1. Meteorology and Transport of Wildfire Pollutants

**Figure 4:**
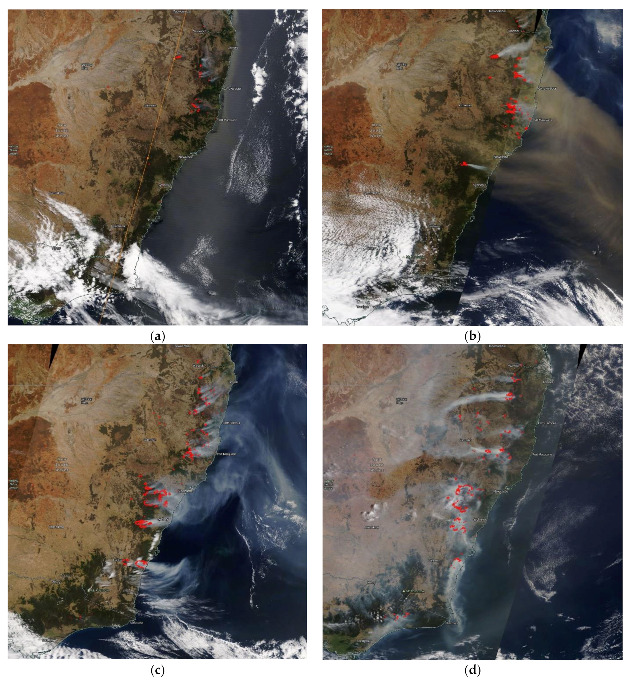

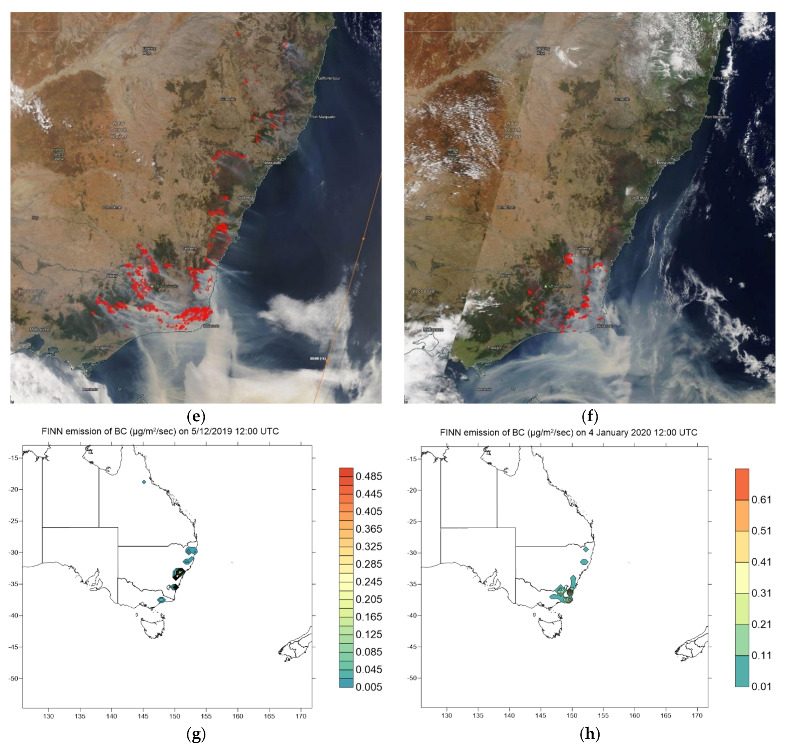
Fires started in northern NSW and the Blue Mountains (northwest of Sydney) as seen by the MODIS Aqua/Terra satellite on 6 November 2019 (**a**) and 7 November 2019 (**b**). From early December to the end of the month, fires were more intense in the Blue Mountains and the south coast: 5 December 2019 (**c**), 18 December 2019 (**d**). By early January, fires were mainly in the south coast and the border region with Victoria on 4 January 2020 (**e**) and the East Gippsland (Victoria) toward the end of January 31 January 2020 (**f**). Emission of BC (black carbon) as a marker of wildfires from the FINN on 5 December 2019, 12:00 UTC, (**g**) and on 4 January 2020, 12:00 UTC (**h**).

The wildfires started in late October, November mainly in northern NSW and the Blue Mountains. In 2020, Filkov et al. [25] reported that the Gospers Mountain fire in the Blue Mountains started on 26 October 2019 while the fires in northern NSW started in the beginning of November and lasted for more than a month. Remote sensing data provided an overall view of the beginnings of the fires, their progress and endings. The MODIS Aqua/Terra satellite data of hot spots and smoke plumes reveal the extent of the wildfires from early November to early December as shown in Figure 4. 

From the remote sensing data and simulation results based on the meteorology and air quality dispersion model WRF-Chem, the progress of the Black Summer 2019–2020 wildfires across southeastern Australia in terms of meteorological drivers, emissions and transport of air pollutants from wildfire sources is summarized below.

#### 3.1.1. November and December 2019 Period

**Figure 5:**
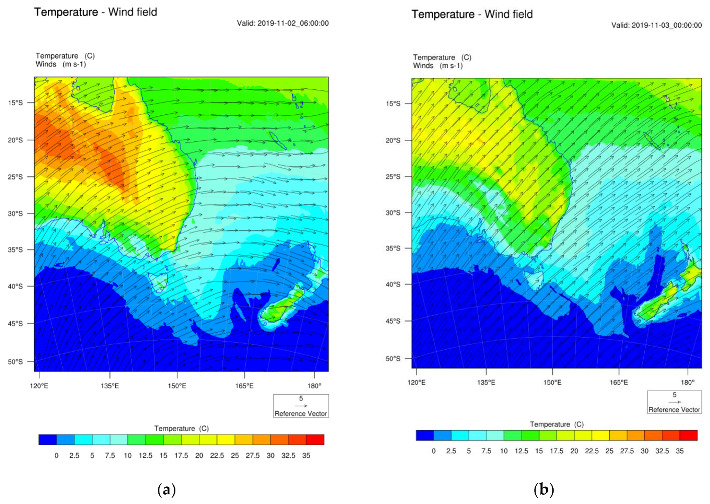

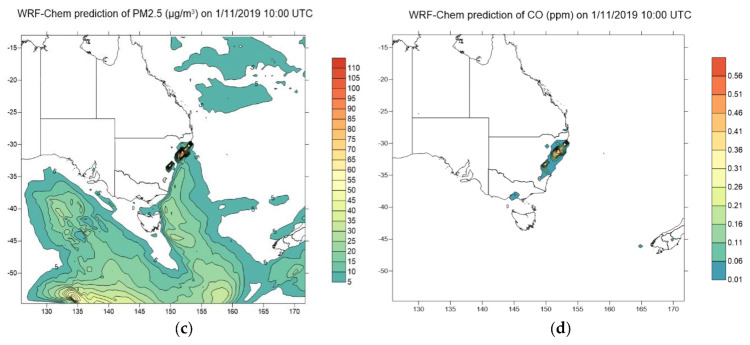
Predicted surface wind and temperature on (**a**) 2 November 2019, 06:00 UTC, and (**b**) 3 November 2019, 00:00 UTC. (**c**) Predicted PM_2.5_ on 1 November 2019, 10:00 UTC, with marine aerosols dominating over the coast and offshore. (**d**) CO concentration on the ground with a high concentration to the southwest of the fires on 1 November 2019 at 10:00 UTC.

**Figure A1:**
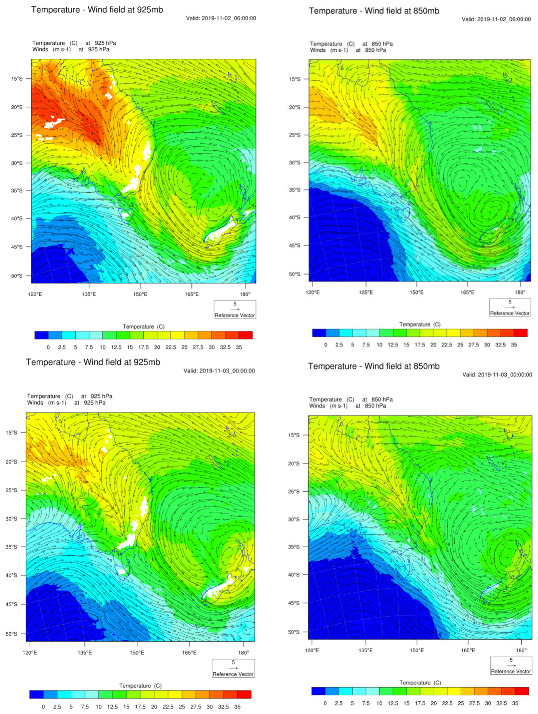
Predicted wind and temperature at 925 mb and 850 mb on 2 November 2019, 06:00 UTC, and 3 November 2019, 00:00 UTC.

Wildfires started in the first week of November in northern NSW. But it was not until 7 November 2029 that the effect on air quality became severe in the nearby coastal town of Port Macquarie for the following 16 days when peak PM_2.5_ concentrations were above 100 µg/m^3^ (>1000 µg/m^3^ for 2 days). The predicted surface wind pattern for the first week of November (as shown in Figure 5) showed a southeasterly wind flow across the east coast of Australia, hence, it dispersed the pollutants offshore. However, upper wind patterns at 925 mb, 850 mb (as shown in Figure A1 in the Appendix A) were different from those on the surface. The plume after being injected into the upper atmosphere drifted down south above the ground and then intruded down causing high concentration of pollutants such as PM_2.5_ on the ground as shown in Figure 5c. On 1–5 December 2019, the wind pattern at the ground level and above the ground was mostly southwesterly from the Southern Ocean and, after crossing the mountains of the Great Dividing Range, would become more westerly. This can be seen by the direction of smoke plumes as detected by the MODIS Aqua/Terra satellites shown in Figure 4c and the fire location corresponds with the FINN emission spatial pattern of BC in Figure 4g. The animation clip of the wind flow and PM_2.5_ concentration from 1 December 2019, 00:00 UTC, to 5 December 2019, 00:00 UTC, is provided in the Supplementary Materials. 

#### 3.1.2. January 2020 Period

Simulation using WRF-Chem for the period from 01/01/2020 to 08/01/2020 based on FINN emission fluxes from fires detected by satellites as hot spots shows the pattern of air pollutants dispersion in eastern Australia. The wildfire emission pattern corresponds with the hot spots as detected by MODIS Aqua/Terra satellites as shown in Figure 4e,f. The predicted surface wind fields over the simulation period of 1 to 8 January 2020 showed that at the beginning, from 1 January 2020, 00:00 UTC, to 3 January 2020, 07:00 UTC, moderate southwesterly wind at ~5 m/s flowed over Victoria and NSW and gradually changed to westerly wind over Victoria and then NSW from 3 January 2020, 16:00 UTC. The westerly and northwesterly wind then dominated from 4 January 2020, 16:00 UTC, to 5 January 2020, 14:00 UTC, over the eastern Australia domain. From 5 January 2020, 16:00 UTC, to 7 January, 08:00 UTC, more westerly wind bringing hot air from inland to the coast which was already subsumed under intense fires. The upper air movement was more complex with air circulation occasionally occurred to and from the coast and is not shown here. Figure S3 in the Supplementary Materials shows some of the surface wind on selected days. 

The AOD as predicted using the WRF-Chem simulation on 3 January 2019 in this study is compared with the AOD as predicted using a global model called WACCM (Whole Atmosphere Community Climate Model) from the NCAR ACOM (Atmospheric Chemistry Observations and Modeling) group in the US. The AOD predictions from the WRF-Chem and WACCM models were remarkably similar. This was not surprising as both models used the same fire emission data from the FINN. The AOD prediction also corresponded well with the AOD observation as measured by MODIS Terra/Aqua satellites (see Figure S4 of the Supplementary Materials). 

The markers for wildfires are the emissions of carbon monoxide (CO) and black carbon (BC). The prediction of ground concentration of these species from the WRF-Chem simulation on 3 January 2019 showed the wildfires were concentrated in the east coast of Victoria near the NSW border. The simulation results on this day for PM_2.5_ also showed the contribution of dust aerosols from the dust storm in central Australia to the ground PM_2.5_ concentration loading in addition to the smoke from the wildfires (see Figure S5 and S5c in the Supplementary Materials). 

**Figure 6:**
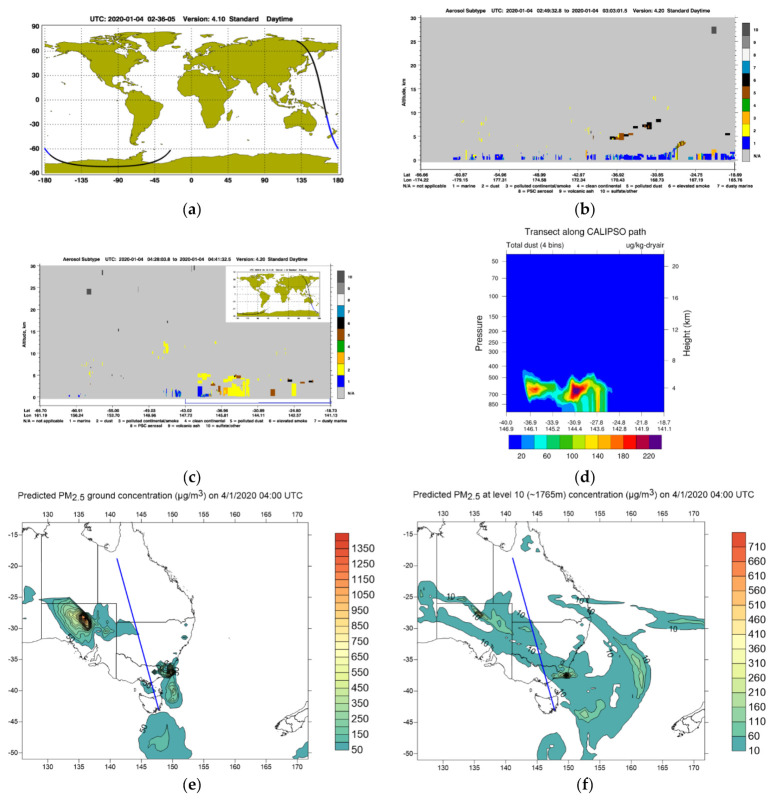
CALIPSO satellite path (**a**) and aerosol profile (**b**) over the Coral Sea and New Zealand on 4 January 2020 at 2:36 UTC. The CALIPSO aerosol profile (**c**) and WRF-Chem prediction of transect of dust along the satellite path (**d**) and spatial distribution of PM_2.5_ concentration at the ground (**e**) and the height ~1765 m (**f**) on 4 January 2020, 4:00 UTC. CALIPSO aerosol profile identifies 10 aerosol subtypes: marine, dust, polluted continental/smoke, clean continental, polluted dust, elevated smoke, dusty marine, PSC (polar stratospheric cloud) aerosols, volcanic ash and sulfate/others.

The dispersion of smoke and dust plumes into the atmosphere allowed the pollutants to be transported farther from the emission sources. Aerosol layers formed from the dispersion and transport of smoke in the atmosphere could be studied using the WRF-Chem simulation. Observation data from the CALIPSO lidar’s vertical profile were used to verify the prediction from WRF-Chem in the upper atmosphere. Figure 6 shows the CALIPSO aerosol profile on 4 January 2020 at 2:36 UTC and 4:14 UTC and the WRF-Chem-predicted PM_2.5_ concentration along the satellite path. The CALIPSO satellite path on 4 January 2020 at 4:14 UTC passed above central Australia and the satellite’s aerosol vertical profile showed thick dust layers above the dust sources in the Lake Eyre Basin (LEB). This showed that the dust event in central Australia happened in early January 2020 when the wildfires on the east coast of Australia were at their peak before subsiding toward late January 2020. WRF-Chem simulation used the Air Force Weather Agency (AFWA) GOCART dust emission scheme and the prediction of dust concentration corresponded reasonably well with the CALIPSO observations. 

**Figure A2:**
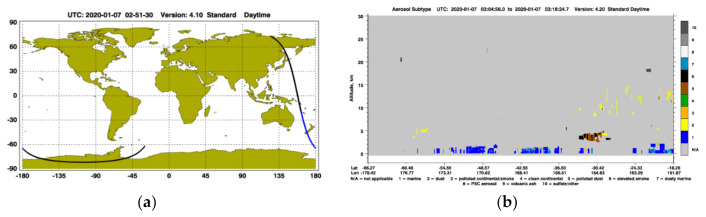
CALIPSO satellite path (**a**) and aerosol profile (**b**) over the Coral Sea and New Zealand on 7 January 2020 at 2:51 UTC. Smoke aerosols (black) over the Tasman Sea in the troposphere at the height of ~3 km to ~5 km.

**Figure A3:**
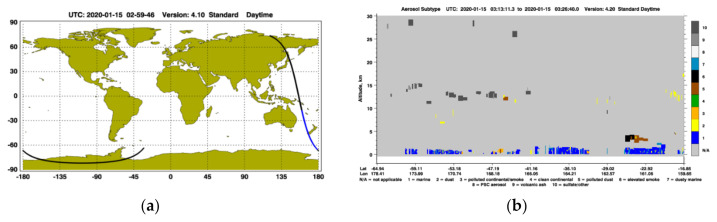
CALIPSO lidar’s profile of aerosols on 15 January 2020 along the CALIPSO satellite path from southern New Zealand to the Tasman Sea and the Coral Sea off Queensland (**a**) and distribution of aerosol types above the ground (**b**). Smoke aerosols (black) in the upper troposphere (10–15 km) and the stratosphere (25–30 km) over the Tasman Sea and southern New Zealand.

Similarly, for the CALIPSO profile on 7 January 2020, the WRF-Chem prediction was able to capture the vertical distribution of smoke aerosols. By this time, the wildfires subsided on the ground and the worst of the fires were over. Figure A2 of Appendix A shows the vertical profile on 7 January 2020. Dispersion of smoke aerosols at the peak of the wildfires from earlier January to mid-January as shown by the CALIPSO satellite lidar’s vertical profile in the Tasman Sea between Australia and New Zealand on 15 January 2020 was so extensive that the smoke aerosols penetrated into the upper troposphere at 15 km and into the stratosphere at ~25–30 km south of New Zealand. Figure A3 in Appendix A shows the lidar profile of different types of aerosols on the satellite path in the Tasman Sea on this date. The smoke layers at the upper troposphere height would travel farther beyond New Zealand.

The agreement between the prediction of PM_2.5_ dispersion as well as spatial patterns due to wildfires with observation from satellites shows that the WRF-Chem model with the FINN fire emission data could be used to predict the effect of wildfires on air quality such as the PM_2.5_ concentration across NSW and hence the impact on health due to population exposure of air pollutants could be estimated. 

### 3.2. Effect on Air Quality

The soil moisture data provided by the Australian Landscape Water Balance AWRA-L (Australia Water Resource Assessment) model (root zone and upper soil moisture) and the vegetation cover data from the CSIRO from October to February were used to determine the fire risk. The spatial extent of the wildfires could be ascertained using the MODIS Terra/Aqua hot spots and the AOD and CALIPSO lidar satellite data. The air quality data from the DPIE’s air quality monitoring network was analyzed to assess the impact of wildfires on air quality and health. The time series of temperature and relative humidity in the GMR from 2019 December to January 2020 showed that when there was very high temperature, the relative humidity was usually low. Figure S6 in the Supplementary Materials shows the time series of temperature and relative humidity measured at Randwick and Liverpool. On 19 December 2019, the peak temperature reached 38.8 °C and 41.1 °C in Randwick and Liverpool and the relative humidity was very low at 18.2% and 12.5%, respectively. Similarly, on 31 December 2019, 4 January 2020 and 23 January 2020, peak temperatures measured at these sites were accompanied by low humidity. 

**Figure 7:**
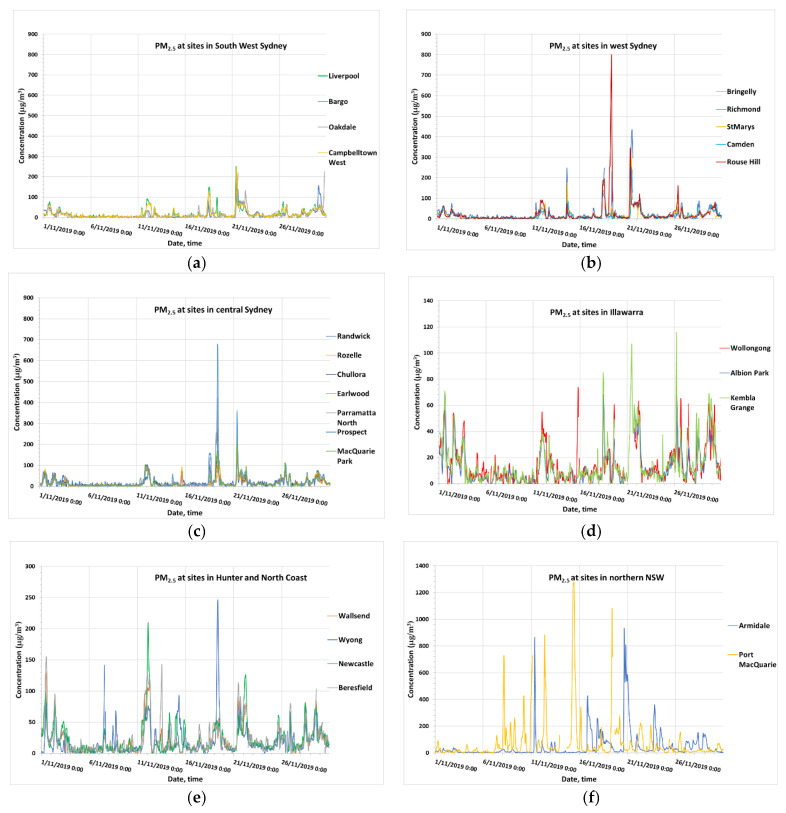
PM_2.5_ concentration at monitoring sites in South West Sydney (**a**), west Sydney (**b**), central Sydney (**c**), Illawarra (**d**), Hunter (**e**) and northern NSW (**f**) from 1 November 2019 to 30 November 2019.

With low soil moisture, high temperature and low humidity, the risk of fires increased. In northern NSW and in the Blue Mountains west of Sydney, Central Coast, wildfires continued from 6 November 2019 until 4–7 December 2019 when fires were more intense and widespread. In November, fires were intense and widespread in northern NSW and caused a very high concentration of PM_2.5_ as measured at Armidale and Port Macquarie monitoring stations with the peak concentration of 1269.3 µg/m^3^ on 15 November 2019 at Port Macquarie and 917.6 µg/m^3^ on 20 November 2019 at Armidale as shown in Figure 7. Elsewhere, fires in the Blue Mountains caused a high concentration in west Sydney and central Sydney with the peak concentration of 790 µg/m^3^ on 19 November 2019 at Rouse Hill and 677 µg/m^3^ on 19 November 2019 at Prospect, respectively, but elsewhere the peak PM_2.5_ concentration was only about 250 µg/m^3^ in South Western Sydney (Liverpool and Campbelltown West), 245 µg/m^3^ in Central Coast and Lower Hunter (Wyong) and about 100 µg/m^3^ in Illawarra (Kembla Grange). Illawarra was the least affected region in November. 

**Figure 8:**
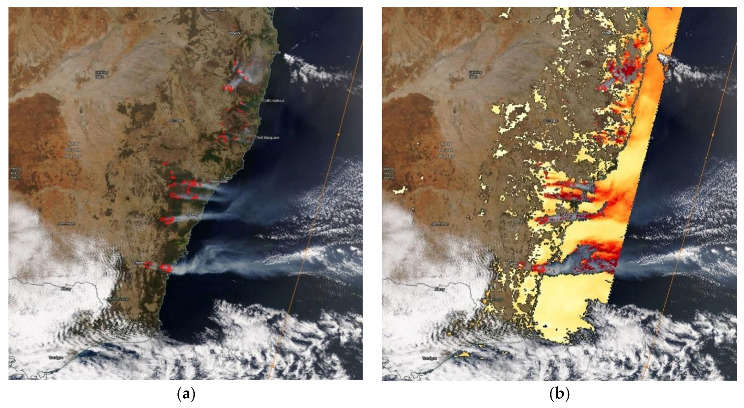
MODIS Terra/Aqua thermal hot spots image on 3 December 2019, 00:00 UTC, (**a**) and the AOD image from the MODIS Terra satellite, land and ocean (**b**).

From early December, the fires caused an elevated concentration of PM_2.5_ up to 273.7 µg/m^3^ (6 December 2019, 08:00) as measured at Port Macquarie. High PM_2.5_ concentrations from 200 µg/m^3^ up to above 400 µg/m^3^ were measured at a number of stations in Sydney (Bringelly, Camden, Liverpool, Oakdale, Campbelltown West, Rozelle, Randwick, Parramatta North, Chullora, Earlwood, Macquarie Park and Prospect) and in the Lower Hunter–Central Coast region (Wallsend, Newcastle, Beresfield and Wyong) from 3 December 2019 to 6 December 2019. Figure 8 shows a MODIS Aqua/Terra satellite image of the fires and the AOD (aerosol optical depth) caused by the fires on 3 December 2019.

**Figure 9:**
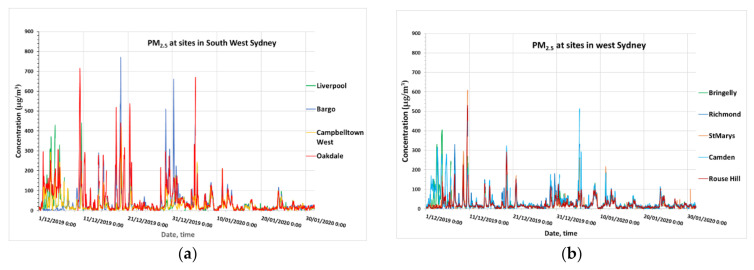

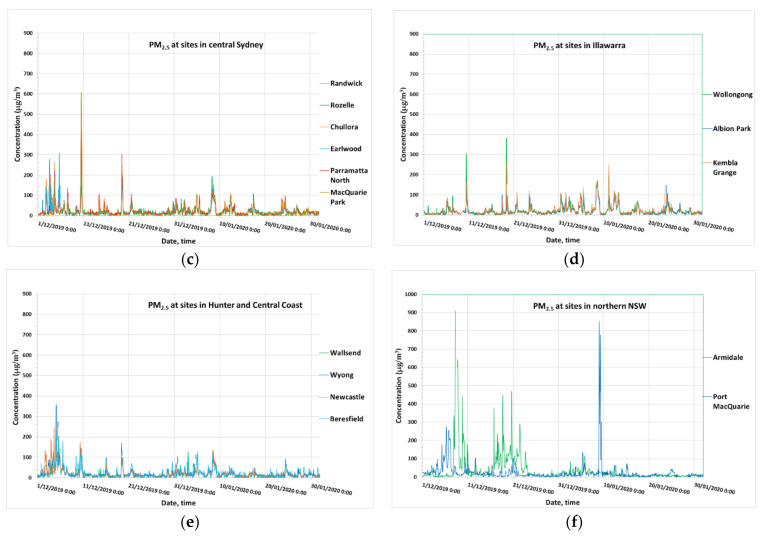
PM_2.5_ concentration at sites in South West Sydney (**a**), west Sydney (**b**), central Sydney (**c**), Illawarra (**d**), Hunter (**e**) and northern NSW (**f**) from 1 December 2019 to 31 January 2020.

Figure 9 shows the time series of PM_2.5_ from 1 December 2019 to 30 January 2020 as measured at the monitoring sites in the GMR of Sydney and northern NSW. On 10 December 2019, at Rozelle, the PM_2.5_ concentration was 533.5 µg/m^3^ at 13:00, at Macquarie Park, 610.6 µg/m^3^ at 12:00, at St Marys, 608.7 µg/m^3^ at 13:00, at Rouse Hill, 531.6 µg/m^3^ at 12:00 and at Wollongong, 299.7 µg/m^3^ at 10:00. The concentrations were then falling until 19 December 2019 when peak PM_2.5_ concentrations were detected at level about and above 300 µg/m^3^ at Parramatta North, Macquarie Park, Camden, Rouse Hill, Wollongong, Kembla Grange. A High concentration on 29, 30 and 31 December 2019 from 300 to above 600 µg/m^3^ also occurred at Bargo. 

In January 2020, high PM_2.5_ concentrations were detected at Camden, St Marys, Oakdale and Kembla Grange on 5 January 2020, at Randwick, Wollongong, Kembla Grange and Albion Park on 8 January 2020 and at Wollongong, Kembla Grange and Albion Park on 10, 11 and 12 January 2020. From early January, the wildfires were more intense in the south coast of NSW and in eastern and northwestern Victoria.

Like PM_2.5_, ozone follows a similar pattern described herein. In early December and mid-December, high ozone concentrations were detected in Lower Hunter and Sydney, then from early January, when the wildfires shifted to the south, Illawarra detected elevated concentration of ozone. Figure S7 in the Supplementary Materials shows the ozone concentration time series during December 2019 and January 2020 at Sydney, Lower Hunter and Illawarra sites. Ozone levels exceeded the NSW air quality goal (10 pphm) at many sites during the wildfire period. In Randwick, ozone was 11.2 pphm on 5 December 2019, 14:00, a level that had not been seen at this long-term site since the 1970s. On 10 December 2019, the levels were 17.9 (12:00), 17.6 (12:00), 15.7 (12:00), 15.7 (13:00), 14.4 (13:00), 12.3 (13:00), 12 (13:00) and 11.6 pphm (13:00) in Earlwood, Chullora, Liverpool, Parramatta North, Bringelly, St Marys, Bargo and Richmond, respectively. 

On 19 December 2019, peak ozone concentrations were detected at levels of 17.9 (12:00), 15.7 (12:00), 13.2 (11:00), 11.4 (12:00), 15.4 (12:00) and 11 pphm (13:00) in Rozelle, Earlwood, Parramatta North, Liverpool, Chullora and Bringelly, respectively, and on 21 December 2019, high ozone levels of 10.6 pphm (14:00), 10.3 pphm (15:00) and 10.6 pphm (15:00) were observed in Liverpool, Bringelly and Oakdale. High levels of 11.2 (30 December 2019, 15:00), 10.6 (31 December 2019, 10:00), 11.4 (31 January 2020, 17:00), 11.6 (31 January 2020, 16:00) and 10.1 pphm (31 January 2020, 15:00) were detected in Richmond, Oakdale, Bargo, Oakdale and Bringelly, respectively. 

In Lower Hunter and Central Coast, high levels of ozone above 10 pphm were measured in Newcastle (10.2 pphm (5 December 2019, 13:00) and 10.4 pphm (19 December 2019, 12:00)), in Wallsend (10.5 pphm (19 December 2019, 13:00)) and in Beresfield (12.6 pphm (21 December 2019, 15:00)). In Illawarra, a high level of ozone occurred in Wollongong (11.1 pphm (19 December 2019, 13:00)). Overall, 10 December 2019 and 19 December 2019 were the two worst ozone days in the GMR of Sydney.

**Figure 10:**
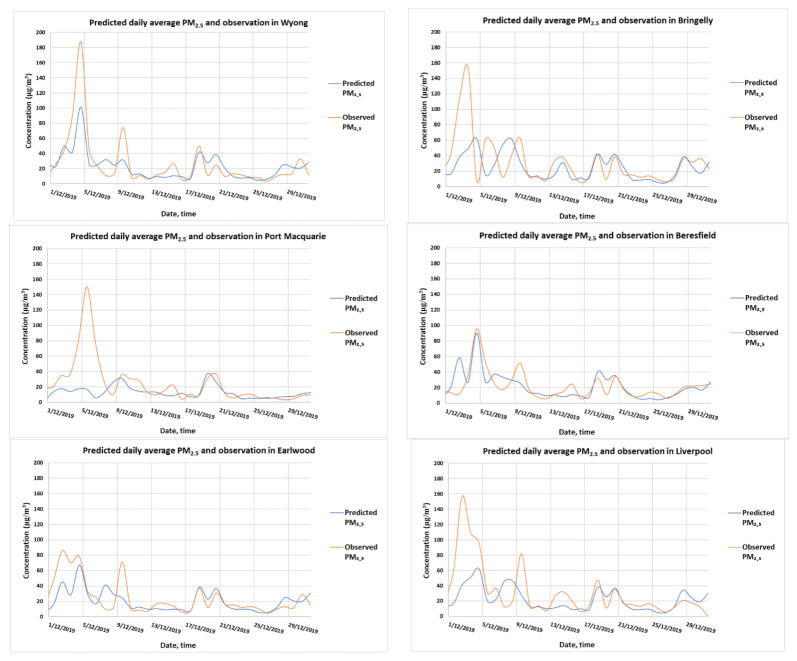

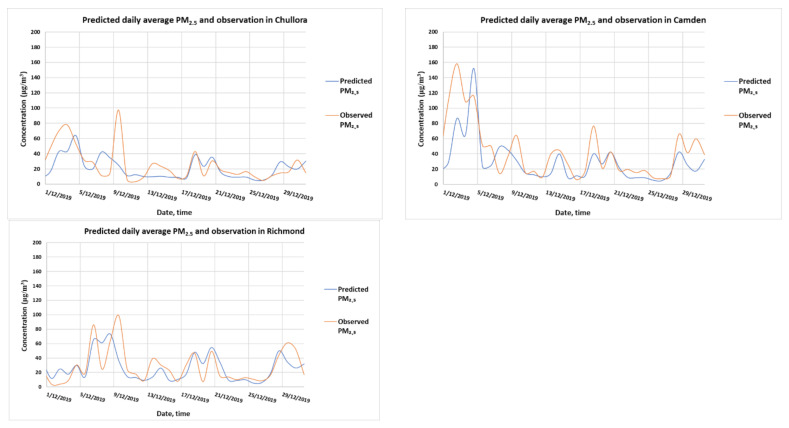
Predicted and observed daily average PM_2.5_ concentrations in Wyong, Bringelly, Port MacQuarie, Beresfield, Earlwood, Liverpool, Chullora, Camden and Richmond.

**Figure A4:**
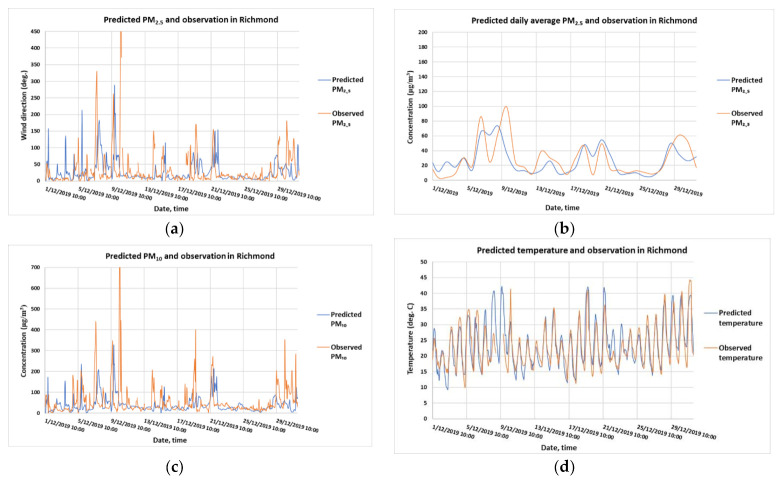

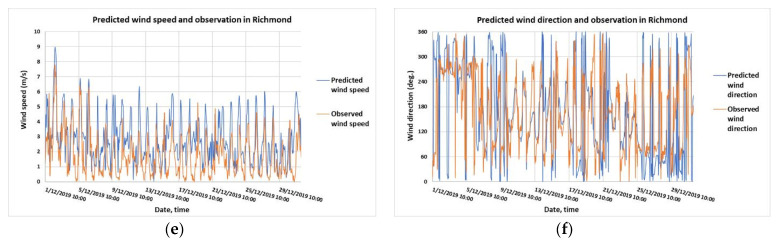
Predicted and observed hourly PM_2.5_ (**a**), daily average PM_2.5_ (**b**), hourly PM_10_ (**c**), temperature (**d**), wind speed (**e**) and wind direction (**f**) at Richmond in the north west of Sydney in December 2019.

Evaluation of the WRF-Chem wildfires simulation using the FINN emission data and the NCEP reanalysis meteorological data sets was performed by comparing some of the predicted meteorological variables such as wind and temperature and the predicted concentration of PM_2.5_ with observed data at the monitoring stations. Figure A4 in the Appendix shows the results of the comparison at a number of sites in the GMR. Considering the 12 km by 12 km grid resolution used in the model, the prediction at hourly intervals as compared with point observations was rather good for temperature and wind direction with temperature correlation and IOA (index of agreement) of ~0.83 and ~0.75, respectively (for three sites, Richmond, Liverpool and Beresfield). The predicted wind speed and PM_2.5_ concentration were also reasonable (R^2^ and IOA from 0.62 to 0.67 and from 0.70 to 0.73, respectively, for wind speed, from 0.2 to 0.25 and from 0.33 to 0.45, respectively, for PM_2.5_). As we used only the predicted daily average of PM_2.5_ to study the impact of 2019–2020 summer wildfires on population health, the predicted PM_2.5_ at the daily scale as compared to the observed daily average was much better than at the hourly scale (R^2^ and IOA from 0.6 to 0.75 and from 0.61 to 0.86, respectively, for daily PM_2.5_). Figure 10 shows the predicted daily average PM_2.5_ as compared to the observations at many sites in NSW.

### 3.3. Population Exposure to Wildfire Particulates and Health Effects

**Figure 11:**
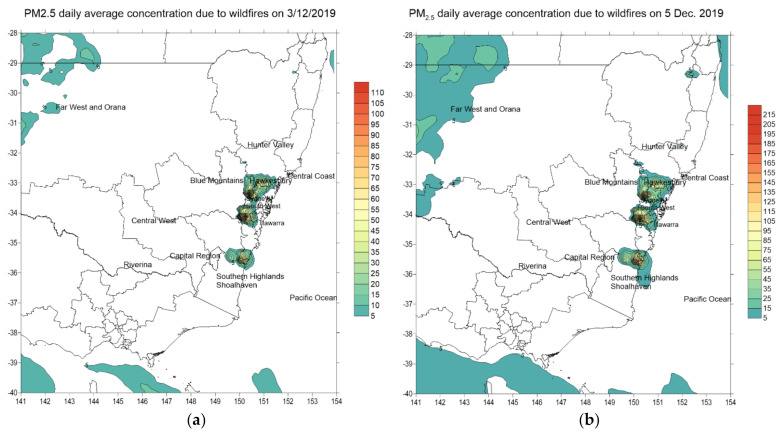

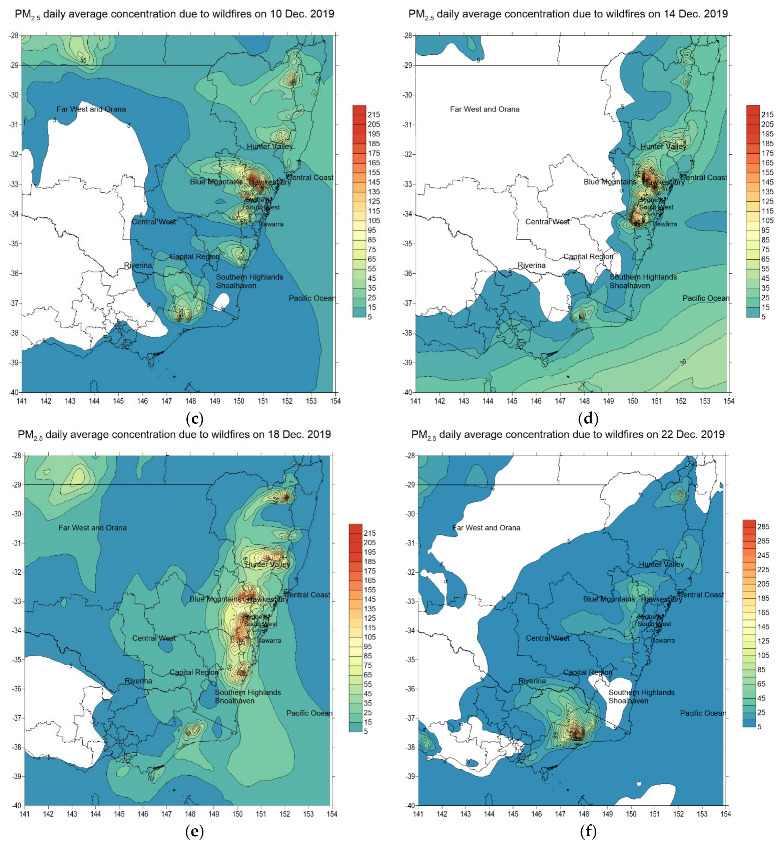

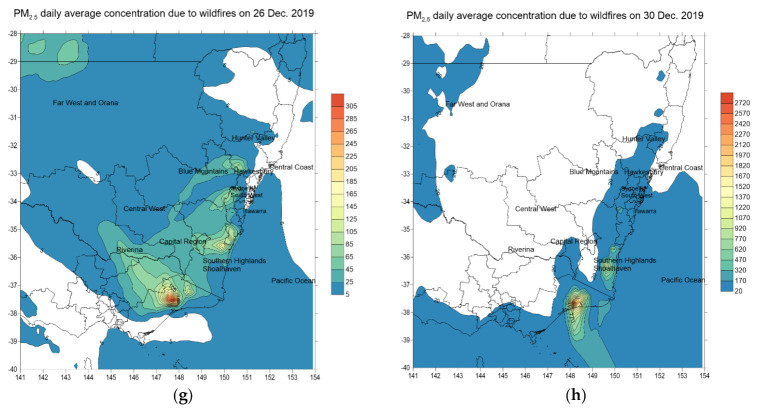
Predicted increase in daily average PM_2.5_ due to wildfires over the Australian Bureau of Statistics (ABS) level 4 census districts (SA4) across southeastern Australia for some selected days in December 2019: 3 (**a**), 5 (**b**), 10 (**c**), 14 (**d**), 18 (**e**), 22 (**f**) 26 (**g**) and 30 (**h**) December 2019. Note: the PM_2.5_ scale is increasing toward the end of December.

The increase in ground concentration of PM_2.5_ in ambient air as a result of wildfires increases the mortality and morbidity rates of the exposed population. The spatial pattern of daily average PM_2.5_ concentration changes with time and space. The peak increases in PM_2.5_ concentration over NSW due to wildfires occurred in early December (7 to 10), mid-December (18 to 21) and toward the end of December 2019 (from 28 to 31 December) with high predicted ground PM_2.5_ concentration as shown in Figure 11. 

**Figure 12:**
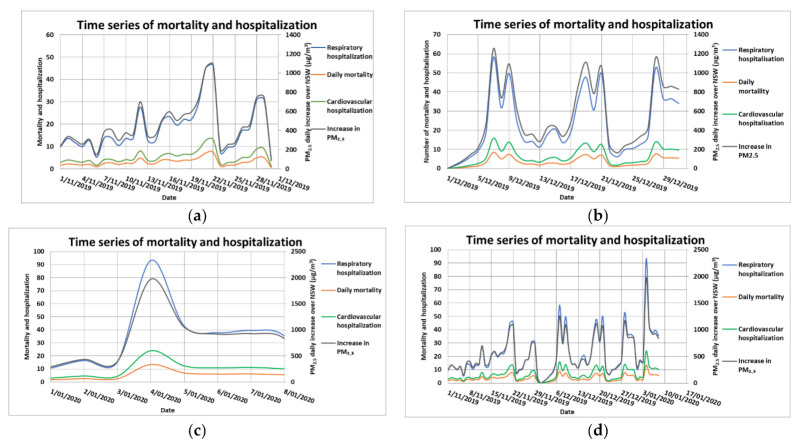
Daily time series of increase in PM_2.5_ over all the census districts (SA4s) in NSW and the rates of mortality and hospitalizations (due cardiovascular and respiratory diseases) due to wildfires in November 2019 (**a**), December 2019 (**b**), 1 to 9 January 2020 (**c**) and in the combined period from 1 November 2019 to 9 January 2020 (**d**).

Figure 11 shows the spatial plots of predicted daily average PM_2.5_ across the states of NSW and Victoria in southeastern Australia due to wildfires for a few selected days in December 2019. The boundaries of the census districts (SA4s) are shown in the map. The corresponding spatial plots for January 2010 are shown in Figure S8 in the Supplementary Materials. Based on Equations (1) and (2) as described in the Methodology section, the effect of an increase in daily average PM_2.5_ concentration in each of the SA4s on mortality, cardiovascular and respiratory diseases hospitalizations was calculated for each day of the wildfires simulation period (from 1 November 2019 to 8 January 2020). Summing up the attributable numbers (ANs) over all SA4s in NSW for each health endpoint gave a daily time series of mortality and hospitalizations as shown in Figure 12 for November, December 2019 (Figure 12a,b) and January 2020 (Figure 12c).

Figure 12d contains time series for the complete simulated period and shows that December 2019 had the most impact on population health. The total mortality estimate for the period from 1 November to 8 January 2020 was about 247 (CI: 89, 409) premature deaths, 437 (CI: 81, 984) cardiovascular diseases hospitalizations and 1535 (CI: 493, 2087) respiratory diseases hospitalizations. In 2020, Borchers Arrigada et al. [9] estimated in their study of the health impact due to this 2019–2020 wildfire event in eastern Australia that for NSW, the mortality, cardiovascular and respiratory hospital admission rates were approximately 219, 577 and 1050, respectively. Their period of study from 1 October 2019 to 10 February 2020 was longer than that in our study. The extra time range as compared with the period in our study is whole of October 2019 and from 11 January to 10 February 2020. The impact of wildfires in the extra time ranges is not significant as the PM_2.5_ concentration due to wildfires was low before 1 November 2019 and after 11 January 2020 when the wildfires subsided significantly. The population-weighted PM_2.5_ levels were below 15 µg/m^3^ in these time ranges as presented in Figure 1 of their study. The results from our study correspond with those of [9] for mortality while our cardiovascular diseases hospitalization estimate is smaller than that estimated in their study, but our respiratory diseases hospitalization rate is higher. 

## 4. Discussion

This study used an air quality modelling tool, WRF-Chem, and the global fire emission data (FINN) derived from remote sensing by satellites to assess the extent of the effect of Black Summer 2019–2020 wildfires on the southeast coast of Australia on air quality and health. Monitoring data from ground-based stations and from satellites were used to corroborate the modelling prediction of meteorological and air quality variables. The prediction from the model allowed understanding the evolution of the effect of the wildfires in terms of pollutant transport and dispersion and assessing the air quality impact, in particular the PM_2.5_ concentration over the modelling domain, in the east coast of Australia where the majority of the population lives. This high spatial resolution prediction of air quality concentration also made it possible to estimate the health impact on population exposure in urban, rural and remote locations to PM_2.5_ from wildfires more accurately than by assigning exposures from central monitoring stations [26]. This is especially important when no monitoring data are available over potentially large geographic areas and hence assuming no spatial variability or interpolation of nearby monitoring datapoints to different areas can introduce an exposure error or misclassification and biased epidemiologic effect estimates [27]. In 2020, Lehtomäki et al. [28] reviewed various studies of mortality from air pollutants (PM_2.5_ and O_3_) and found that there is a wide range of mortality results obtained using different health assessment tools and concentration–response functions in Nordic countries. They reported that sensitivity analysis showed that high spatial resolution is necessary to avoid underestimation of exposure and health effects.

We decided to use WRF-Chem rather than WRF-CMAQ as the high-resolution (1 km by 1 km) daily global FINN emission dataset is available for WRF-Chem with the MOZART chemistry mechanism. The fire radiative power as detected from satellites indicates the intensity of the fire and hence can be scaled to modulate the emission. The emission of pollutants from the flaming and smothering stages of fires is therefore incorporated in the radiative power which the FINN dataset considers. In 2018, Wilkins et al. [20] showed in their study of wildfire effect using ground-based statistical datasets on air quality (ozone and particulates) from 2008 to 2012 in the US using the CMAQ model that the performance of the CMAQ model largely depends on the type of wildfires and emission estimates. In their study, the use of statistical datasets compiled by various organizations featured large uncertainties in emission estimates. We use a single consistent FINN dataset which relies on satellite sensor detection for fires and their radiative power.

Even though the summer 2019–2020 wildfires did not cause as many deaths as the Black Saturday wildfires in Victoria in 2009 or the Ash Wednesday in 1983 in Victoria and South Australia, the costs thereof is much more significant than of those previous wildfires. The costs include the economic loss of properties and businesses in many regional towns and villages and the health impact due to persistent high particle concentration and exposure of people in major population centers over a long period and large affected areas. However, technology also helped people to reduce their exposure to smoke. In 2020, Campbell et al. [29] reported the survey results based on 13% of responses from more than 13,000 people who used AirRater, a free smartphone application that reports air quality and track user’s symptoms in near real-time and helped them to reduce exposure to smoke during the 2019–2020 wildfires. Respondents provided feedback that the application was very useful in helping them to make decisions to avoid or minimize exposure such as staying indoors (76%), rescheduling or planning outdoor activities (64%), changing the location to less affected areas (29%). During the peaks of wildfires, the number of people visiting a general practitioner (GP) actually dropped and the largest decreases in GP attendance claims were seen in the affected regions during the weeks when air quality was recorded as particularly poor and this drop in GP attendance may also have been influenced by health advice to stay indoors [6].

Health alerts to the public from the NSW Department of Health which worked in conjunction with other agencies such as the Department of Planning, Industry and Environment (DPIE) or the Rural Fire Services (RFS) of NSW also helped people to take actions to reduce their exposure to smoke. These interventional actions reduced population exposure and hence theoretically affected the calculation of the estimated exposure and health effects using the method chosen for this study which assumes that the ambient concentration of PM_2.5_ directly and fully affects people. In 2020, Mueller et al. [13] found in their study of ambient air pollution and health effect in northern Thailand including the period when there was intense agricultural biomass burning in March that the risk ratio (RR) between daily PM_10_ and outpatient visits was elevated most on the same day as exposure for chronic lower respiratory disease (CLRD) with RR = 1.020 (95% CI: 1.012 to 1.028) and cerebrovascular disease (CBVD) with RR = 1.020 (95% CI: 1.004 to 1.035), but there was no association with ischemic heart disease (IHD) with RR = 0.994 (95% CI: 0.974 to 1.014). They also found that there was no evidence that the high PM_10_ concentration on biomass burning days showed a clear exposure response effect for CLRD and CBVD visits. They suggested that two possible reasons for this result were that particulates from biomass burning may be less harmful than those from other sources and that at higher PM concentrations identified as occurring due to biomass burning, the risk decreases. But the other factor that can influence the results is that people during episodic events of pollution seek shelter or take measure to reduce their exposure to harmful pollutants in the ambient air. 

In addition to uncertainty in RR for each health endpoint, another source of uncertainty in the estimated results is the uncertainty in the WRF-Chem model. The accuracy of the WRF-Chem air quality model depends on uncertainties of the emission input and the uncertainty of the WRF-Chem model itself (its meteorological and chemical components). Even though the FINN provides the most detailed emission data on both the temporal scale and the spatial scale and its estimates are comparable to other emission datasets such as GFEDv3, the uncertainty assigned to the FINNv1 estimates is about a factor of 2 due to various assumptions such as land cover classifications, estimated burned area, fuel loading and consumption and emission factors [23]. Low-cost sensors could be used to increase the availability of air quality measurement data in areas where standard monitoring network stations are lacking, and modelling output data can then be used to blend with these data using techniques such as the Bayesian method to increase the accuracy of the estimated pollutant concentration for health impact calculations. In 2020, Robinson [30] used low-cost sensor data in addition to the data from the DPIE monitoring station in Armidale (northern NSW) to show the spatial variability of PM_2.5_ and thus help to improve the accuracy of exposure and health impact estimates due to wood heater use during winter in this region. 

Another approach to estimate the PM_2.5_ concentration from wildfires at grid points is the use of machine learning or an AI method instead of the physical modelling method as in this study using WRF-Chem. In 2018, Yao et al. [26] used machine learning to estimate hourly exposure to PM_2.5_ for urban, rural and remote populations during wildfire seasons.

In this study, we only focused on the daily scale effect based on the predicted daily average concentration. However, the health effect of PM_2.5_ exposure during wildfire seasons can be seen on a sub-daily scale as Yao et al. (2020) [14] reported in their study that increased PM_2.5_ concentration was associated with some respiratory and cardiovascular outcomes within 1 h following exposure. Sub-daily scale population health impact analysis can be performed if the accuracy of the current modelling technology to predict the pollutant concentration is much improved. The current WRF-Chem air quality model does not take into account the fire spread and fire behavior on this time scale. The studies and methodology of [31,32] on short- and long-distance fire spotting can be implemented in the air quality modelling system such as WRF-Chem to take into account fire behavior and propagation. 

The other effect of wildfires on people’s welfare-being that should be addressed is the effect on mental health. During the summer 2019–2020 wildfires event, many people became anxious and worried about the wildfires and made calls to the Lifeline crisis support hotline. As a result of this increase in call numbers, a telephone line for people affected by the bushfires was introduced to address the situation [6]. The costs of the Black Summer 2019–2020 wildfires in terms of physical property damage, biodiversity, people health and the economy were significant. Prescribed burning is essential for managing these wildfires. With wildfires increasing in frequency and scope in southeastern Australia and globally [33] due to climate change, in 2020, Morgan et al. [3] found in their study on the history of prescribed burnings in southeastern Australia that even though significant progress of fire and ecosystem science had been achieved in the previous 50 years, the current fire management can neither sustain the full range of ecosystem processes and biodiversity nor reduce to an acceptable level the impact of wildfires on human lives and property. They suggested more investment in training, human capacity and resources to safely and effectively deploy prescribed burning more widely to reduce future wildfire risks and that the potential negative impacts of prescribed burning can be managed effectively with clear communication of the benefits of prescribed burning to the public. However, in 2021, Handmer et al. [34] stressed that for large-scale wildfires, a systemic approach to mitigate and manage fire risks has to be considered. The approach pays special attention to the increased risk of large-scale and extreme fires from tail dependence and spatial dependence. Tail dependence, in which extreme conditions (at the tail of each distribution) such as high temperature and low rainfall can occur concurrently or one extreme condition can enhance the risk of occurrence of the other condition, makes the fires caused by compound events more likely and more extreme. Spatial dependence, on the other hand, reflects the fact that fire risk in one area can be connected with other areas due to extreme dryness occurring everywhere over a large region with available fuel load. Fires over a local area in this situation can ignite or spread to faraway areas through fire spotting or occur simultaneously and then connect to form megafires. Both tail dependence and spatial dependence conditions were present in the Black Summer 2019–2020 wildfires in eastern Australia. There are methods available for assessing the tail dependence and spatial dependence [34]. Importantly, the Black Summer wildfires highlighted the role of climate change on the increasing number and scale of wildfires in eastern Australia and therefore efforts to mitigate the effect of climate change or reduce greenhouse emissions should be considered seriously. 

## 5. Conclusions

The wildfires event in the austral summer of 2019–2020 on the east coast of Australia from late October 2019 to mid-January 2020 was studied in detail using an air quality model and observation data from ground-based and remote sensors. The effect of the wildfires on air quality and population exposure was widespread over New South Wales, Australian Capital Territory and Victoria. Ozone and PM_2.5_ particle levels exceeded the NSW air quality goal at many metropolitan monitoring sites in the Greater Metropolitan Region of Sydney, especially in December. The health effect due to PM_2.5_ as predicted from modeling on the exposed population in each of the ABS census districts (SA4) of New South Wales was estimated based on epidemiological assumptions of the impact function and incidence rates from the 2016 ABS and NSW Department of Health statistical health records. Summing up all SA4 census district results over NSW, we estimated that there were 247 (CI: 89, 409) premature deaths, 437 (CI: 81, 984) cardiovascular diseases hospitalizations and 1535 (CI: 493, 2087) respiratory diseases hospitalizations in NSW over the period from 1 November 2019 to 8 January 2020.

## Data Availability

Data available upon request to the corresponding author.

## Supplementary Materials

The following are available online at https://www.mdpi.com/article/10.3390/ijerph18073538/s1, Figure S1: Upper soil moisture in November, December 2019 and January 2020. Figure S2: Vegetation cover loss from November to December 2019, and from December 2019 to January 2020. Figure S3: Predicted surface wind and temperature. Figure S4: Predicted AOD on 3/1/2020 01:00 UTC as compared with AOD prediction from NCAR ACOM. Figure S5: Predicted concentration of CO, PM2.5, BC (hydrophilic and hydrophobic BC) on 3/1/2020 01:00 UTC. Figure S6: Temperature and relative humidity at Randwick and Liverpool. Figure S7: Ozone concentration at monitoring sites. Figure S8: Predicted daily average PM2.5 across south eastern Australia due to wildfires for some selected days in January 2019 Table S1: WRF-Chem configuration in the namelist.input, Video S1: Simulation of hourly surface wind and PM_2.5_ concentration across eastern Australia from 1 to 5 December 2019.
