# Supplementary material for: The Summer 2019–2020 Wildfires in East Coast Australia and Their Impacts on Air Quality and Health in New South Wales, Australia
Source: Int J Environ Res Public Health. 2021 Mar 29;18(7):3538. doi: 10.3390/ijerph18073538 (PMC8038035; doi:10.3390/ijerph18073538)
Supplement: Supplementary file 1 [file ijerph-18-03538-s001.zip › supplemetary_materials_a_reviewers.docx]

Supplementary Materials.


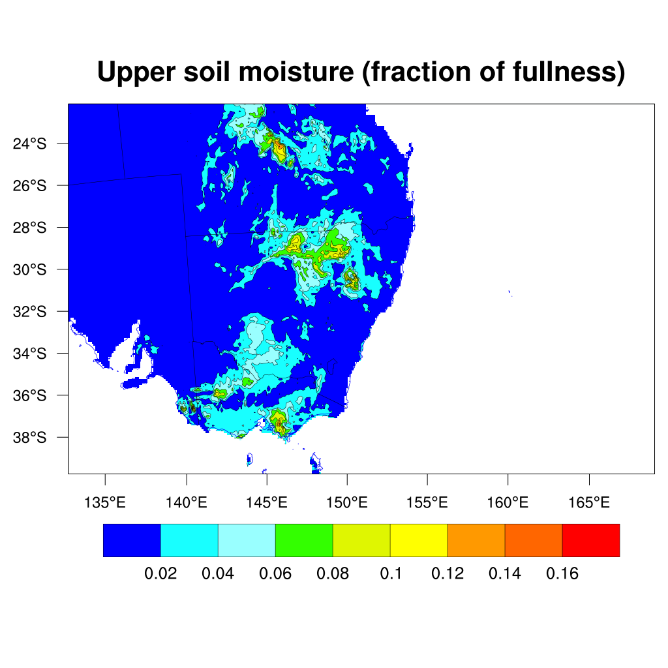

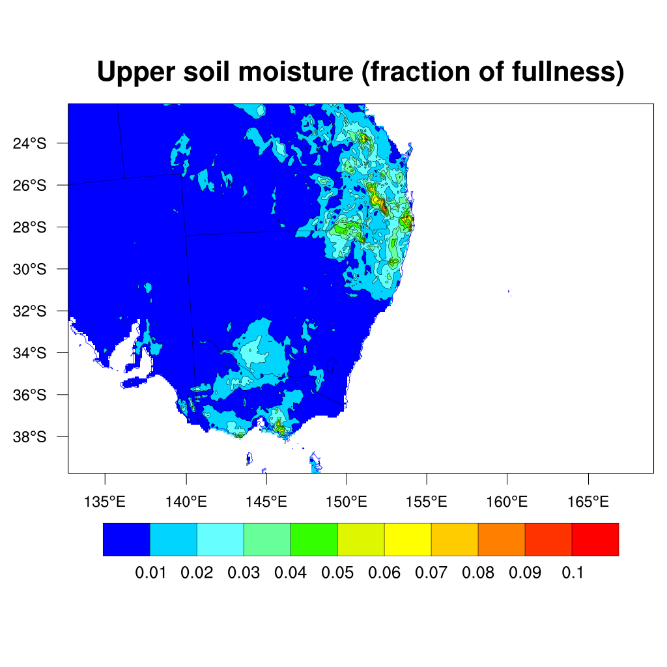


(a) (b)


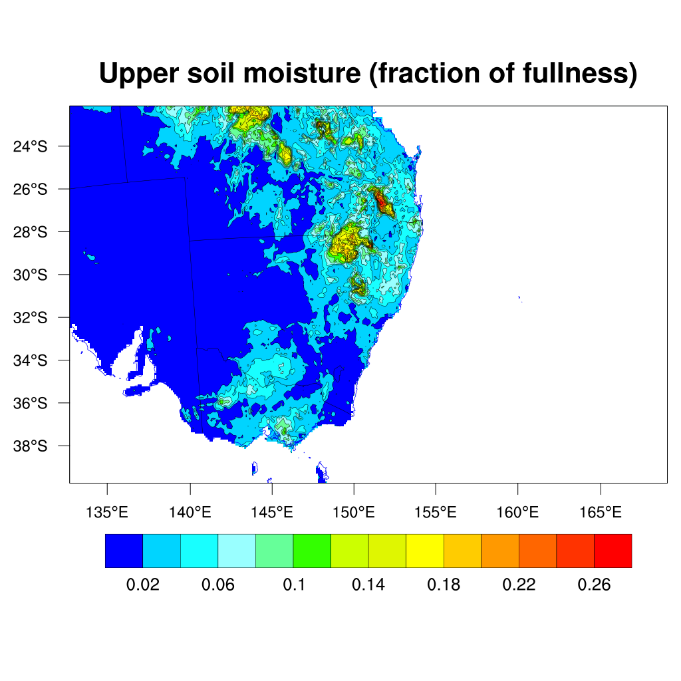

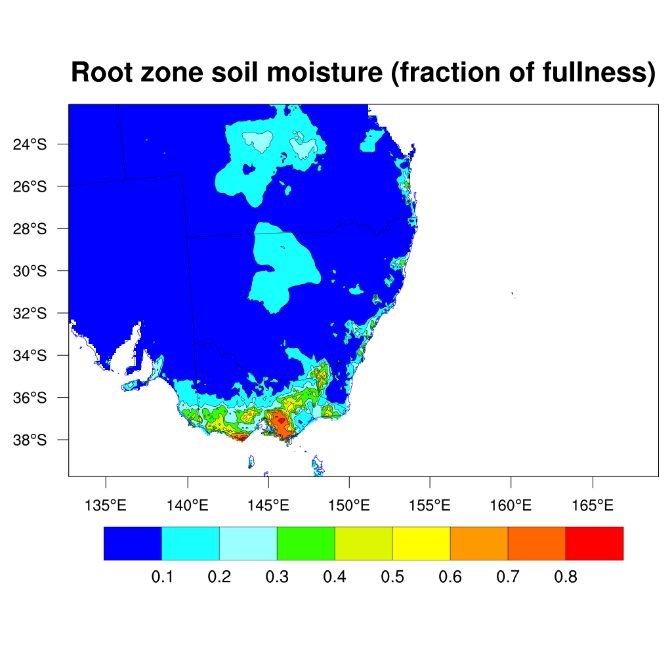


(c) (d)


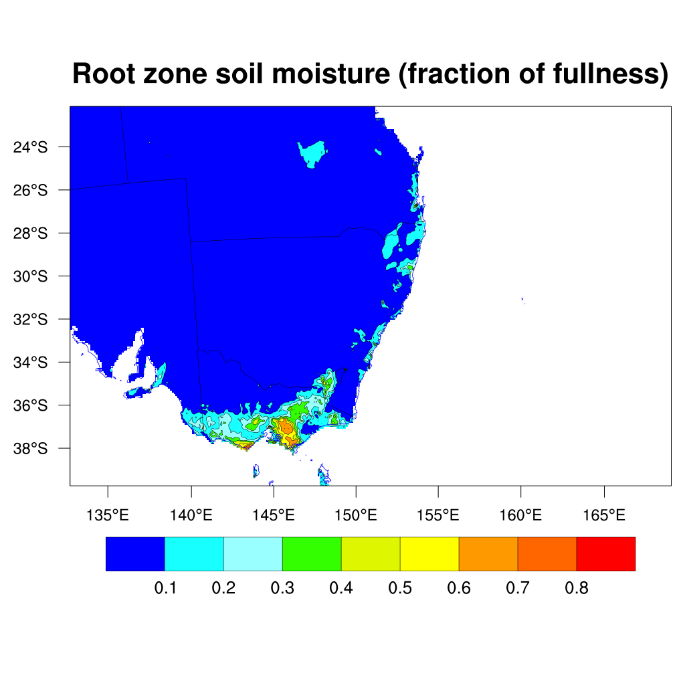

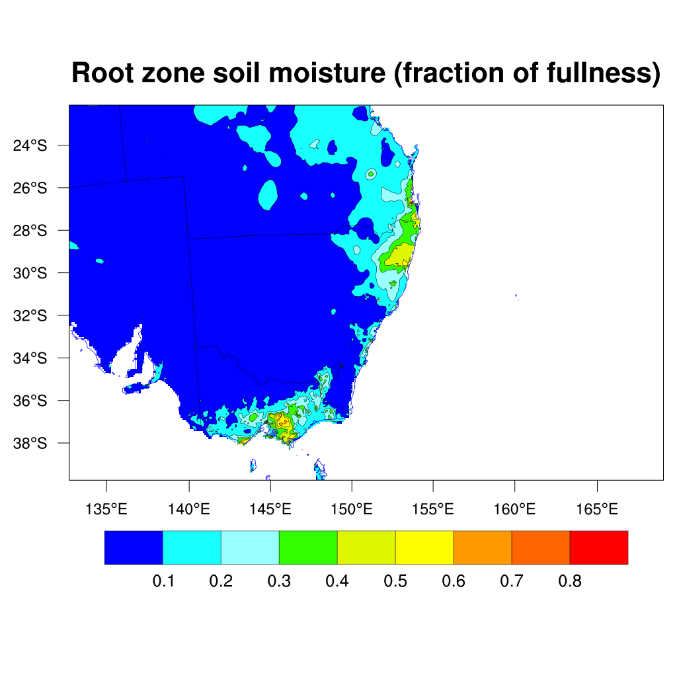


(e) (f)

Figure S1 – Upper soil moisture in November 2019 (a), December 2019 (b) and January 2020 (c). Root zone soil moisture in November 2019 (d), December 2019 (e) and January 2020 (f) (Source: Bureau of Meteorology, Australia Water Resource Assessment (AWRA), Australian Landscape Water Balance. Available online: <http://www.bom.gov.au/water/landscape/#/sm/Actual/day/-28.4/130.4/3/Point////2019/4/10/>, accessed on 17 March 2021)


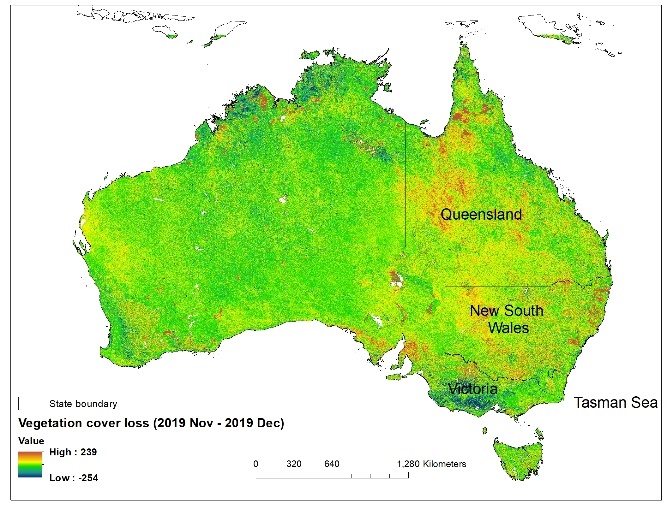

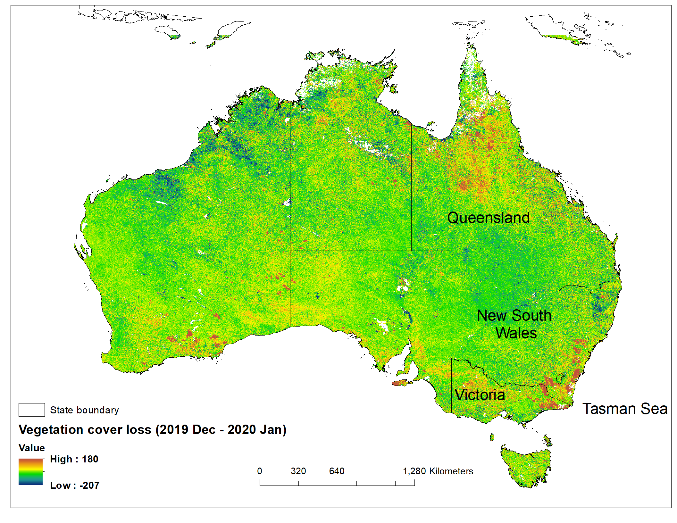


(a) (b)


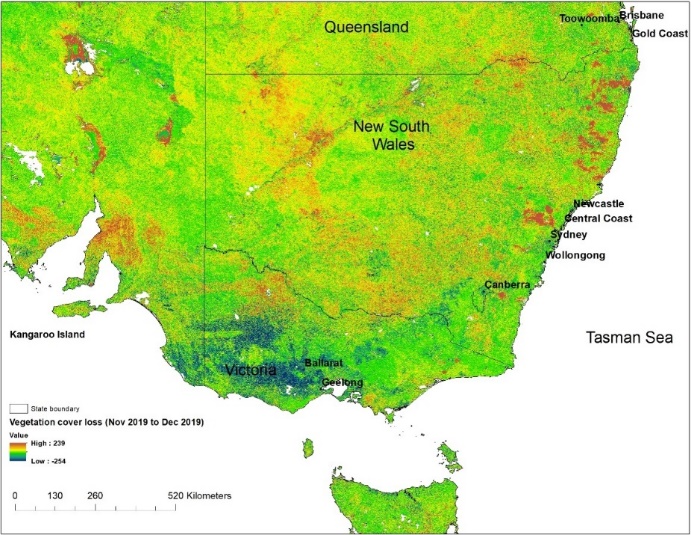

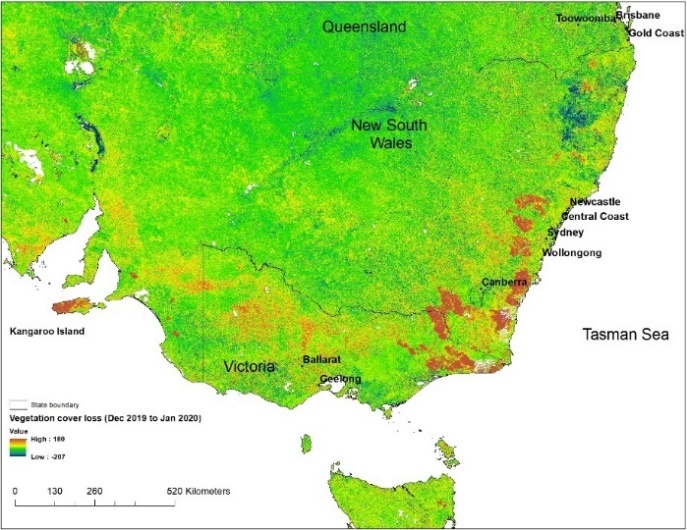


(c) (d)

Figure S2 – Vegetation cover loss from November to December (a) and (c) and from December to January (b) and (d) show the two stages of the summer 2019-2020 wildfires: north coast of NSW, Blue Mountains in November and December 2019 and the south coast toward Victoria, Victorian East Gippsland, the Mallee region west of Bendigo, South Australia and Kangaroo Island, and south Western Australia in January 2020.


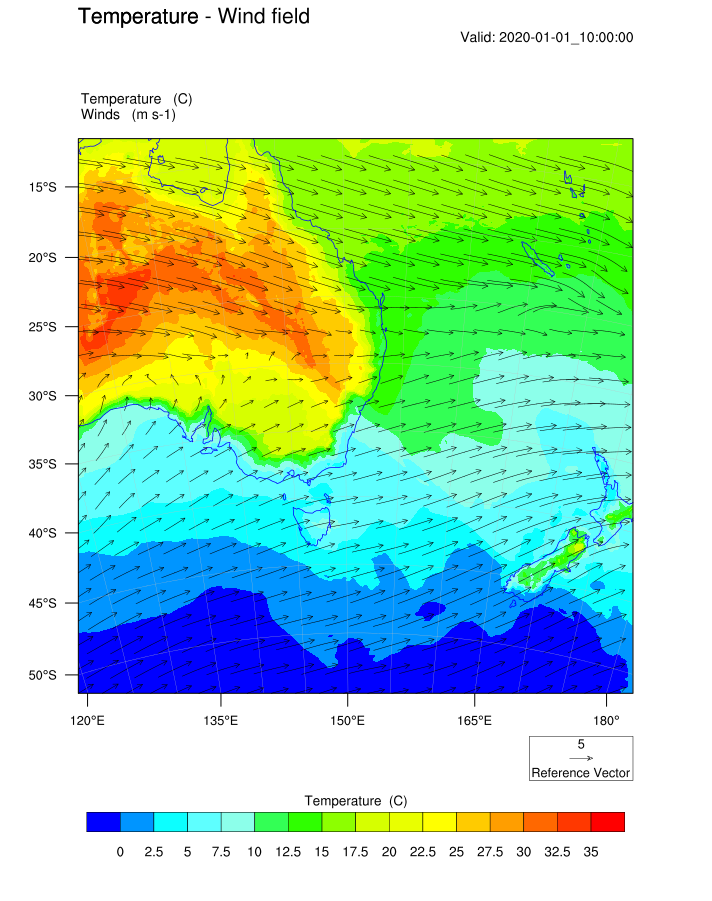

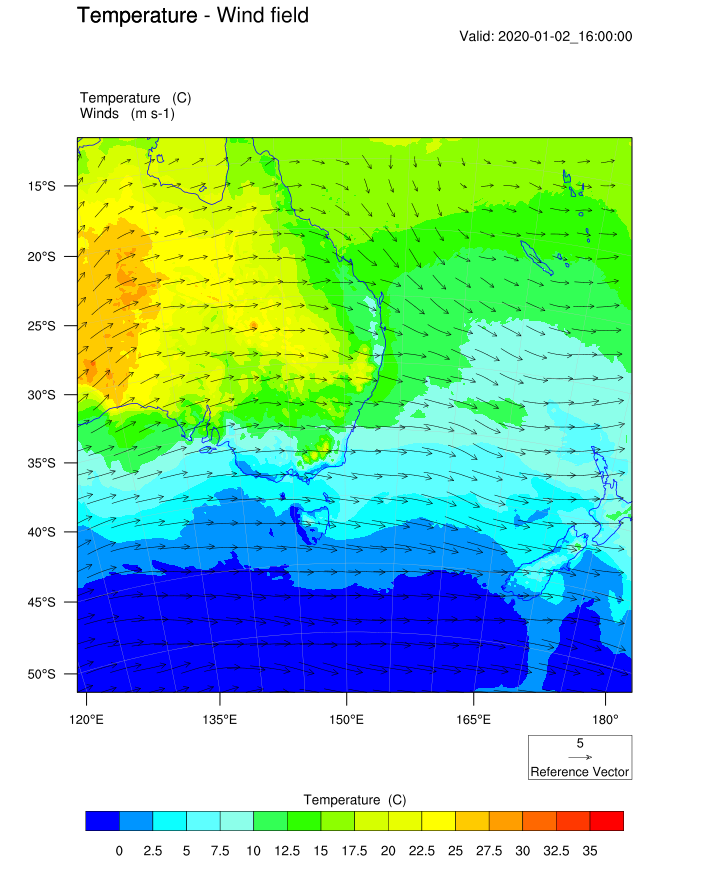


(a) (b)


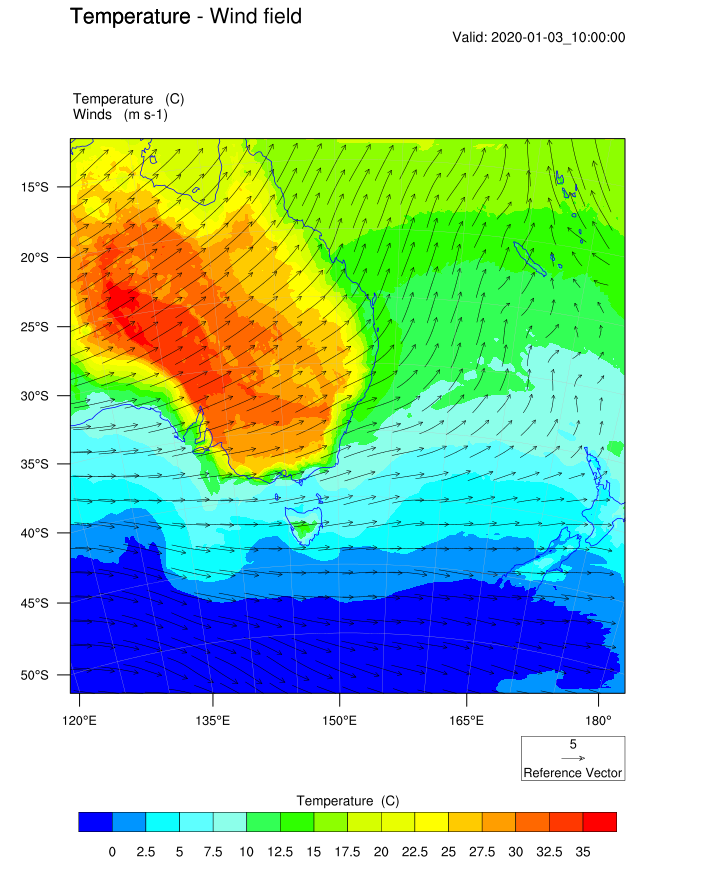

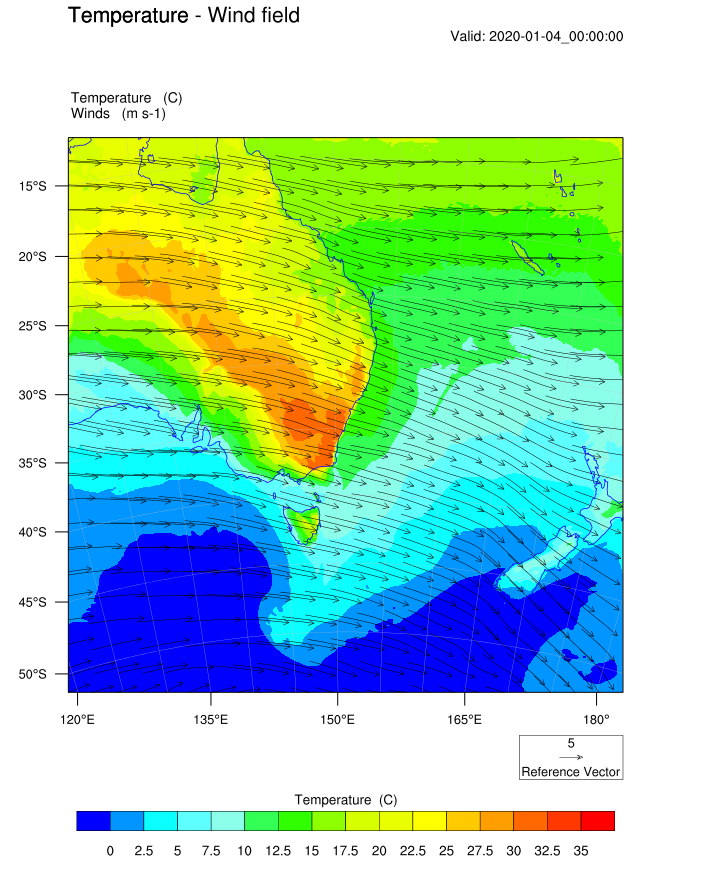


(c) (d)


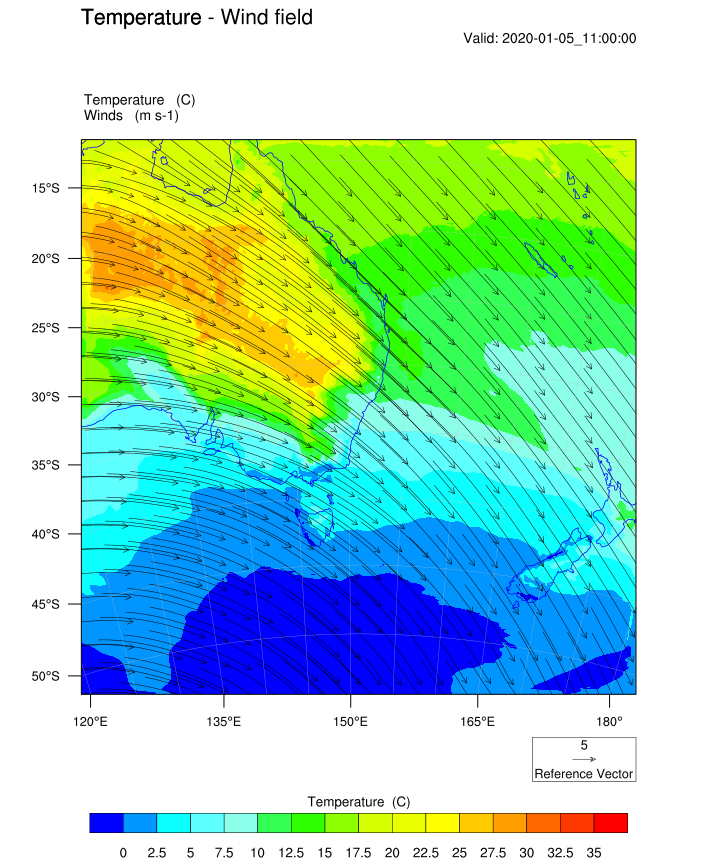

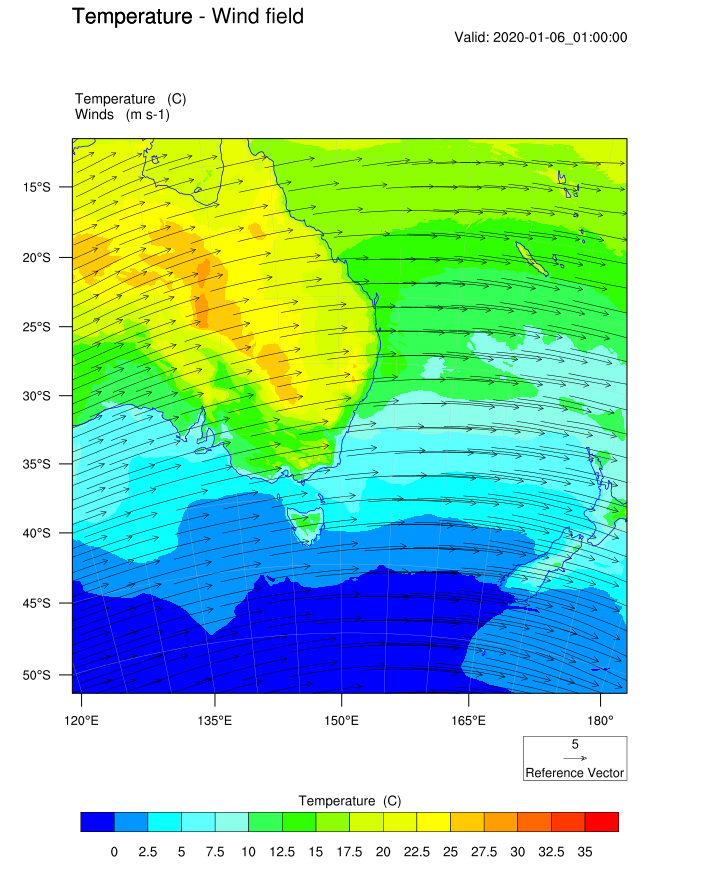


(e) (f)

Figure S3 - Predicted surface wind and temperature on (a) 1 January 2020 10:00 UTC and (b) 2 January 2020 16:00 (c) 3 January 2020 10:00 UTC (d) 4 January 0:00 UTC (e) 5 January 2020 11:00 UTC and (f) 6 January 2020 1:00 UTC

| 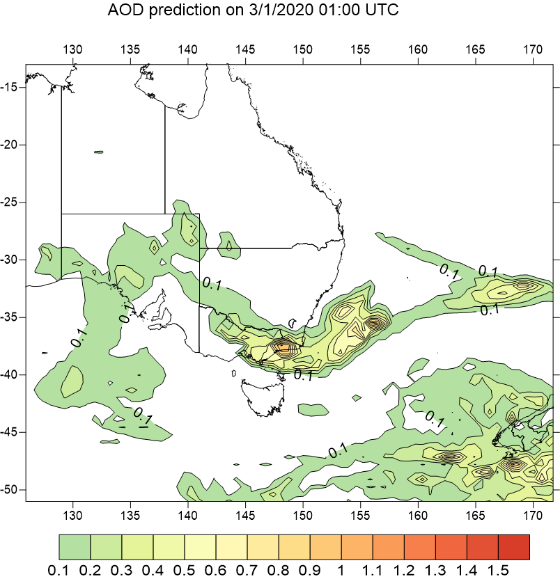  (a) | 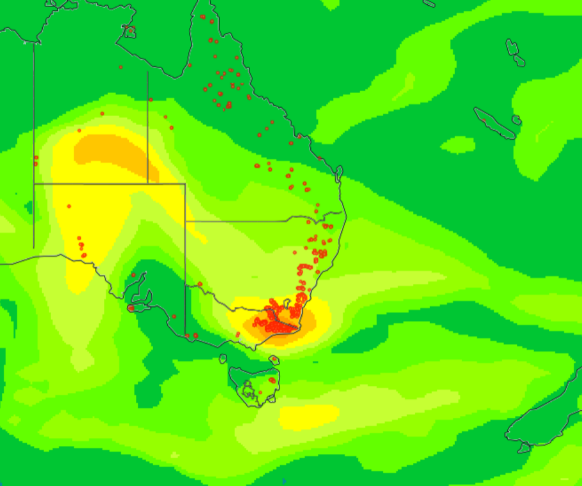  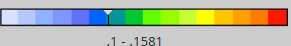  (b) |
| --- | --- |
| 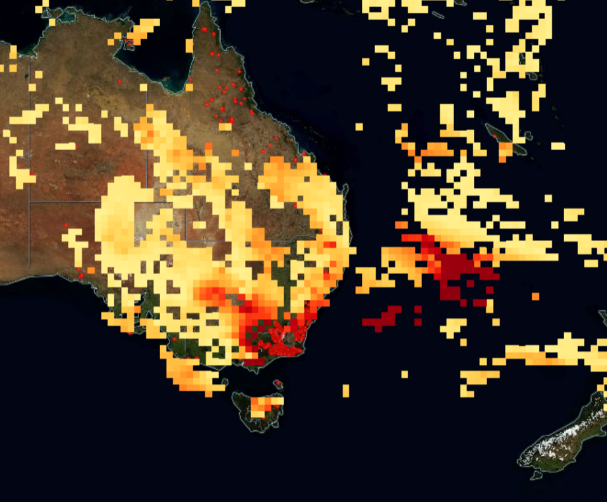  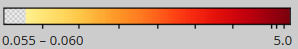  (c) | 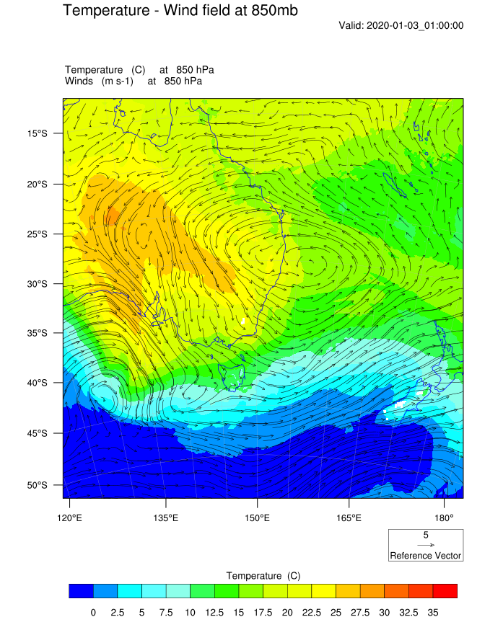  (d) |

Figure S4 – Predicted AOD on 3/1/2020 01:00 UTC (a) as compared with AOD prediction from NCAR ACOM (Atmospheric Chemistry Observations & Modeling) model called WACCM (Whole Atmosphere Community Climate Model), at the same time (source: <https://worldview.acom.ucar.edu/>) (b) AOD as measured and value-added by MODIS combined Aqua/Terra satellite product (c). Wind field at 850mb height (d).


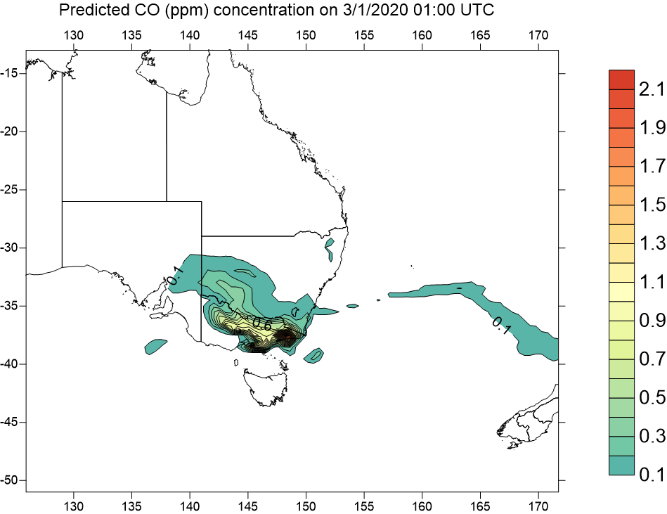

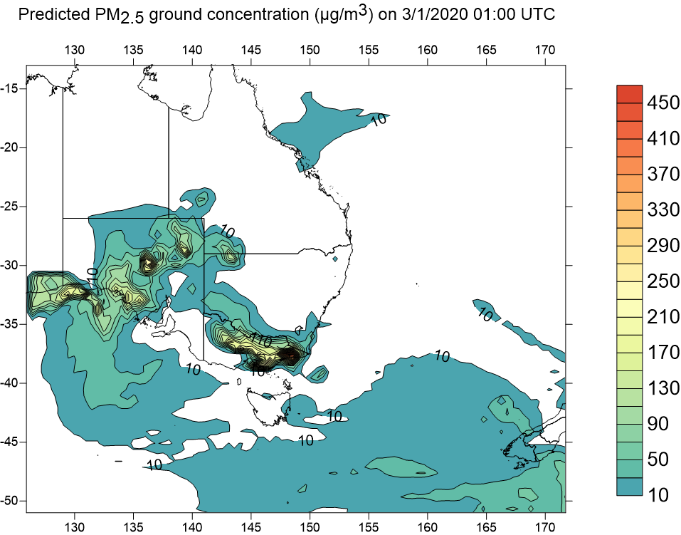


(a) (b)


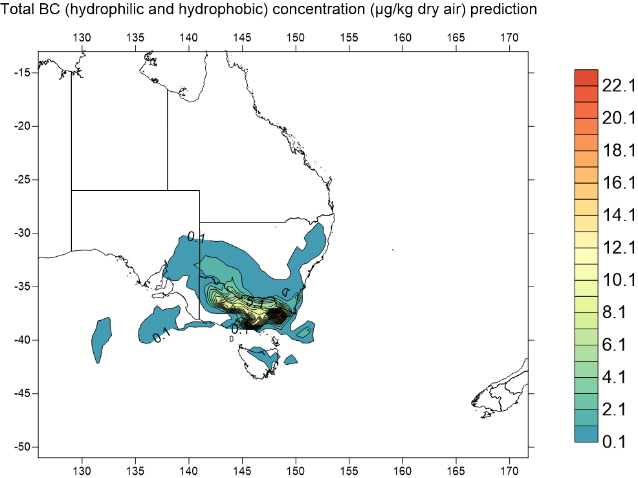


(c)

Figure S5 – Predicted concentration of CO, PM_2.5_, BC (hydrophilic and hydrophobic BC) on 3/1/2020 01:00 UTC. Dust storm also happened in Central Australia as evidence of high PM_2.5_ concentration occurred in South Australia near dust source in Lake Eyre Basin.


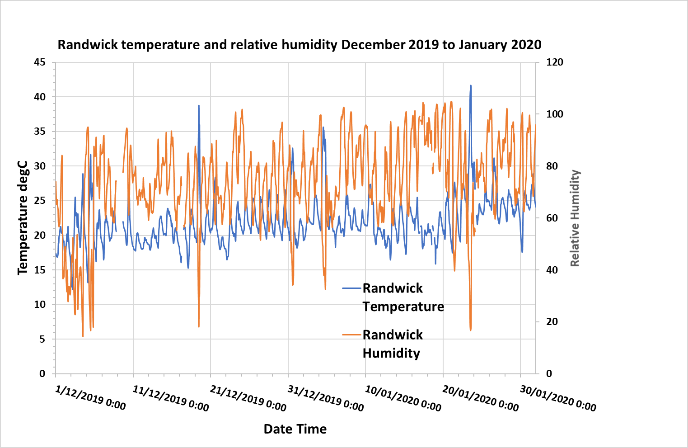

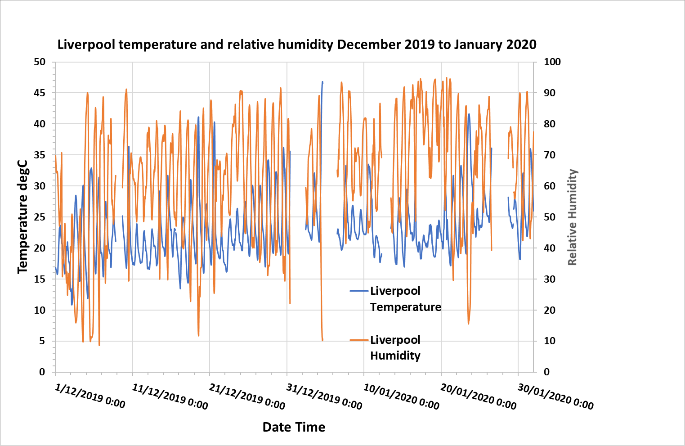


(a) (b)

Figure S6 – Temperature and relative humidity at Randwick and Liverpool.


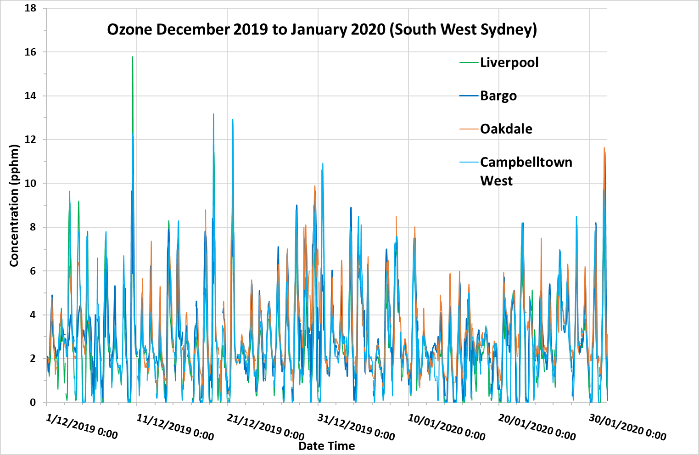

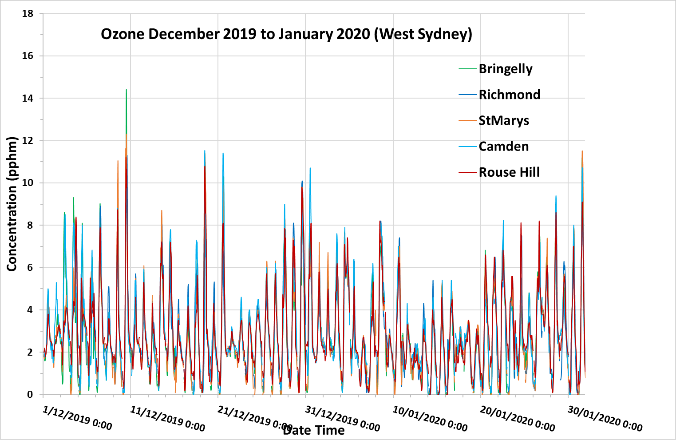


1. (b)


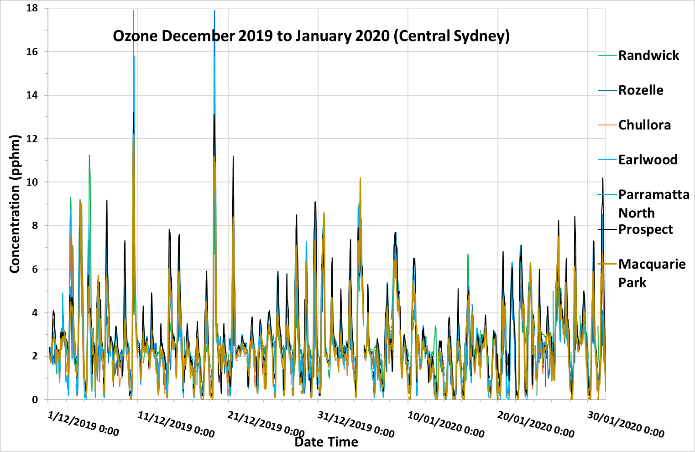

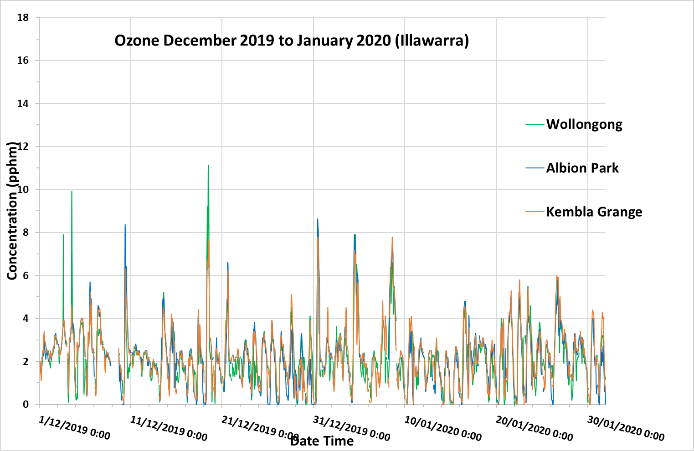


(c) (d)


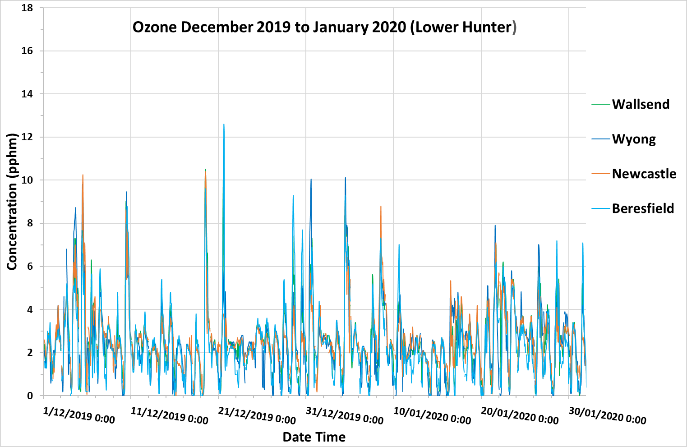

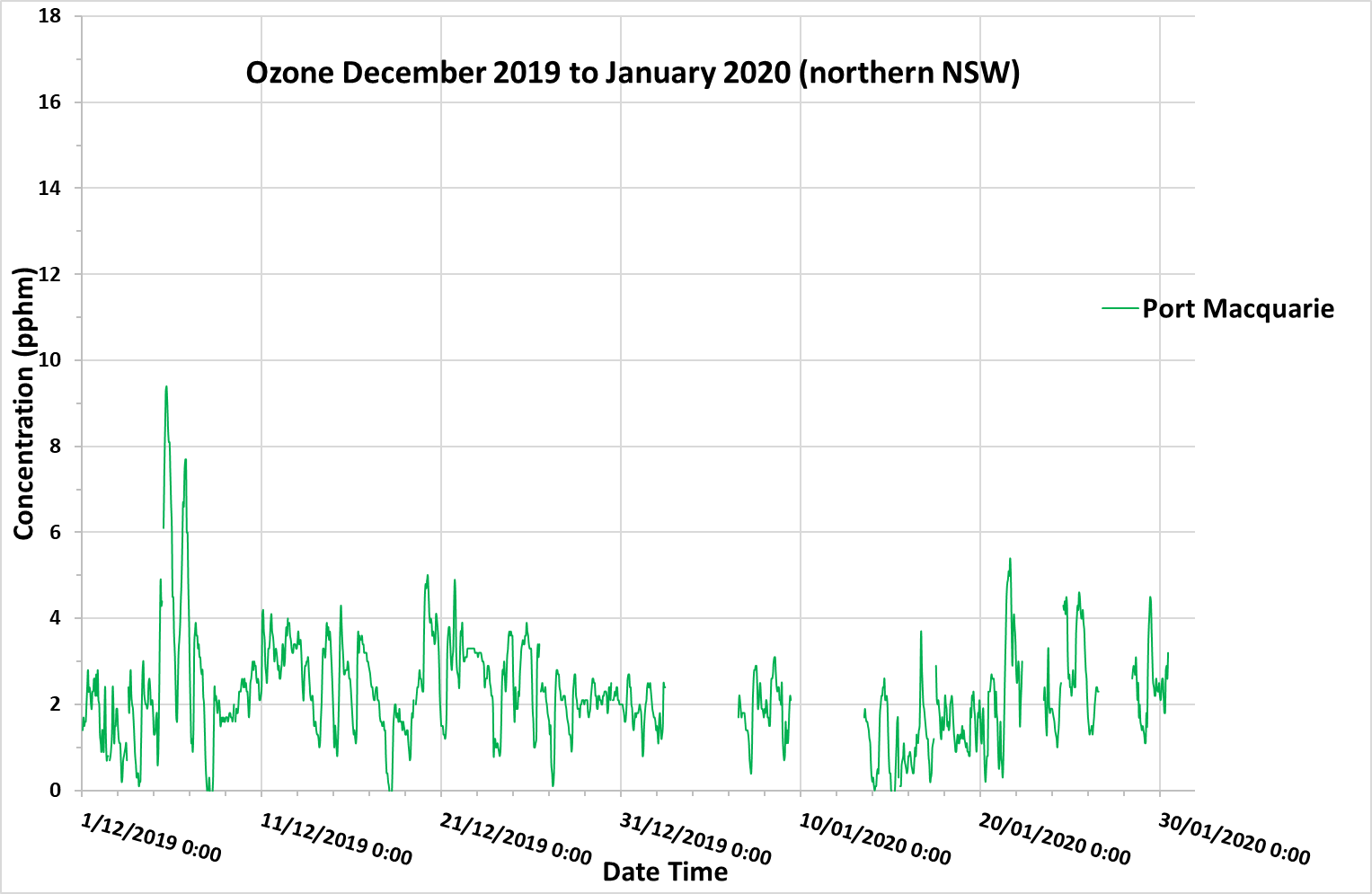


(e) (f)

Figure S7 - Ozone concentration at sites in south west Sydney (a), west Sydney (b), central Sydney (c), Illawarra (d), Hunter (e) and northern NSW (f) from 1 December 2019 to 31 January 2020


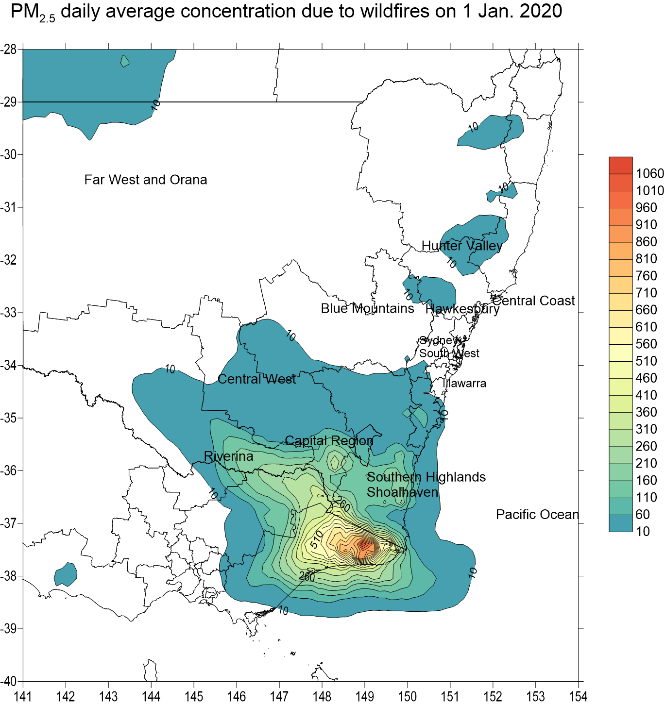

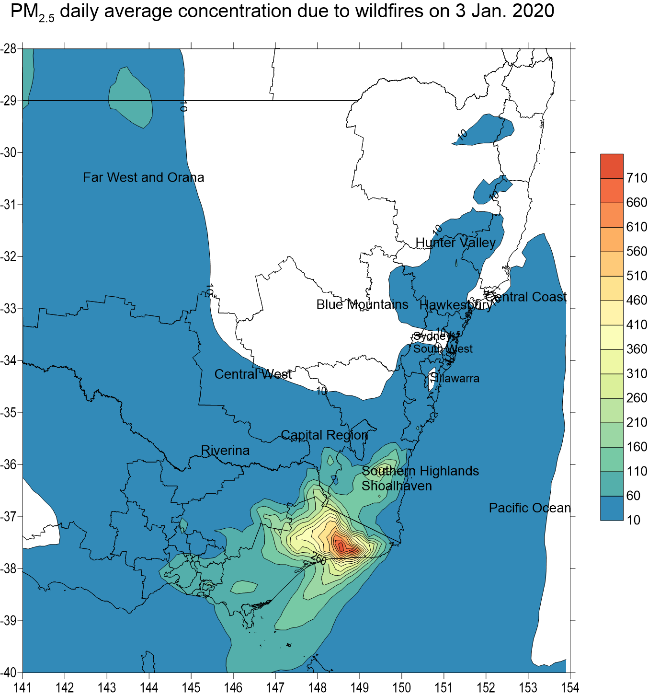


1. (b)


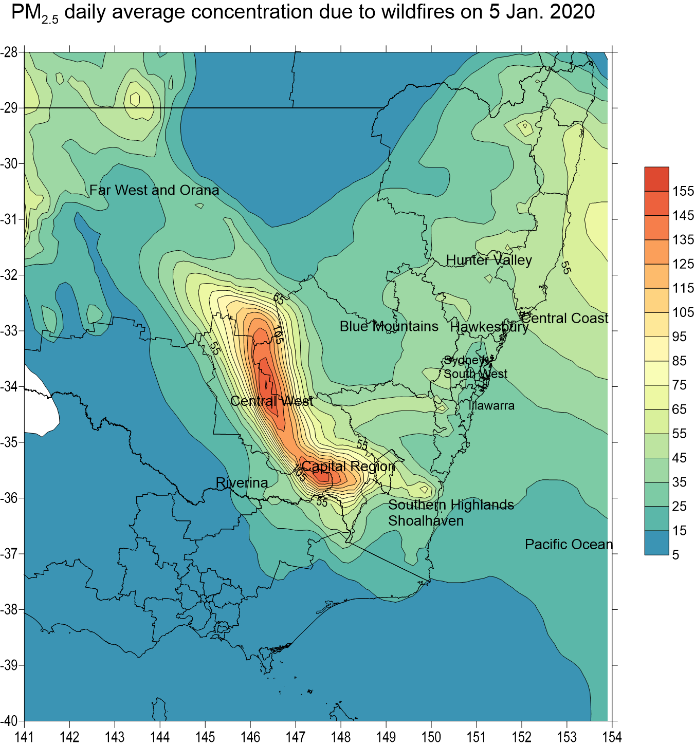

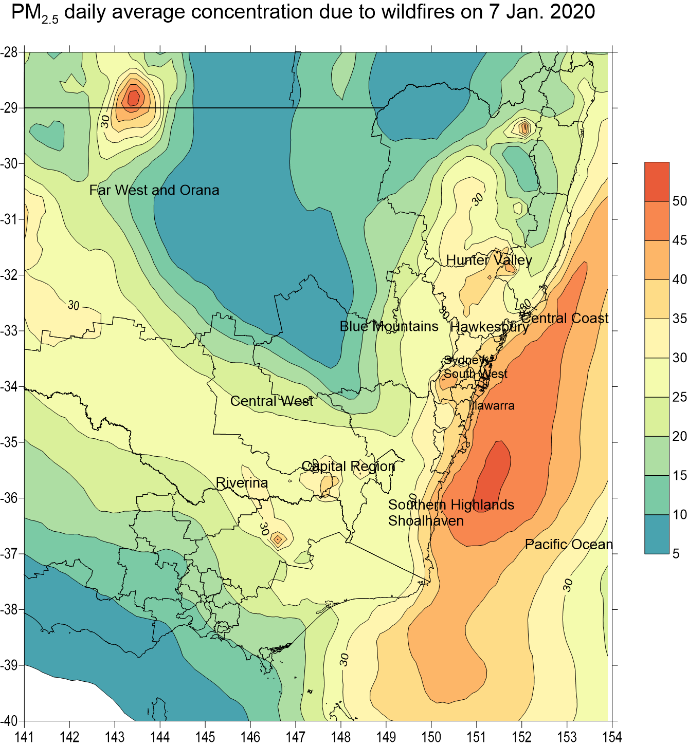


(c) (d)

Figure S8 – Predicted daily average PM_2.5_ across south eastern Australia due to wildfires for some selected days in January 2019: 1 January 2020 (a), 3 January 2020 (b), 5 January 2020 (c) and 7 January 2020 (d)

| Physical parametrisation | Namelist variable | Option | Model/scheme |
| --- | --- | --- | --- |
| Microphysics | mp_physics | 3 | WRF Single Moment |
| Land surface | sf_surface_physics | 2 | Noah Land-Surface Model |
| Surface layer physics | sf_sfclay_physics | 1 | Monin-Obukhov similarity |
| Planetary Boundary Layer | bl_pbl_physics | 1 | YSU scheme |
| Shortwave radiation | ra_sw_physics | 4 | Rapid Radiative Transfer Model (RRTMG) |
| Long wave radiation | ra_lw_physics= | 4 | Rapid Radiative Transfer Model (RRTMG) |
| Cumulus cloud | cu_physics | 1 | Kain-Fritsch scheme |
| Gas/aerosol Chemistry | chem_opt | 112 | MOZART/GOCART |
| Biomass burning option | biomass_burn_opt | 2 | Emission and plume rise for MOZCART |
| Sea salt emission | seas_opt | 1 | GOCART sea salt emission scheme |
| Photolysis | phot_opt | 3 | Madronich F-TUV photolysis |
| Dust scheme | dust_opt | 3 | GOCART-AFWA scheme |
| Aerosol extinction coefficient approximation | aer_opt_opt | 2 | Maxwell-Garnett approximation |
| Aerosol radiative feedback | aer_ra_feedback | 1 | Turn on aerosol radiative feedback with RRTMG model |

Table S1 – WRF-Chem configuration in the namelist.input
