# Supplementary figures and images for: The Summer 2019–2020 Wildfires in East Coast Australia and Their Impacts on Air Quality and Health in New South Wales, Australia
Source: Int J Environ Res Public Health. 2021 Mar 29;18(7):3538. doi: 10.3390/ijerph18073538 (PMC8038035; doi:10.3390/ijerph18073538)

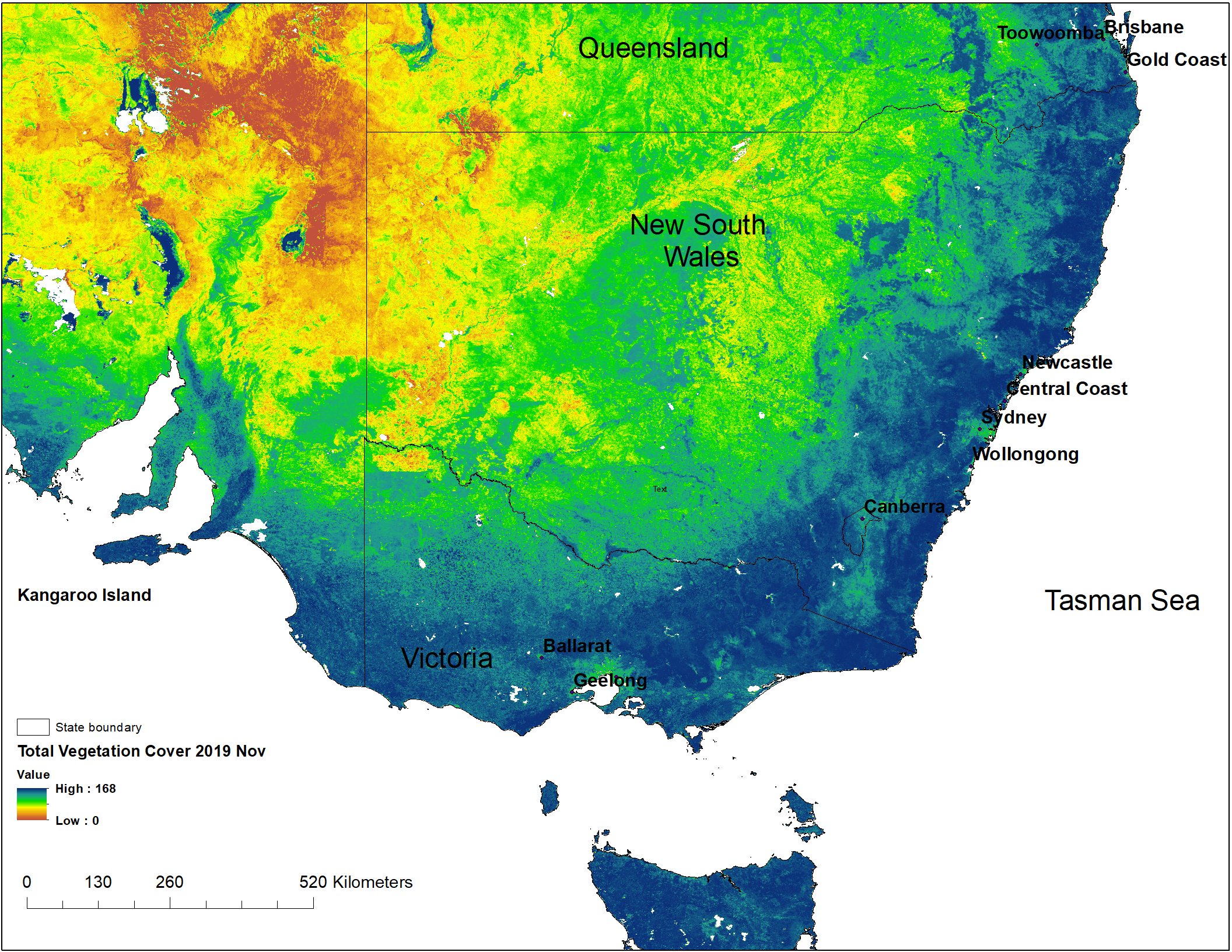

Supplement: Supplementary file 1 [file ijerph-18-03538-s001.zip › vegetation_cover_2019-nov-seaus2.tif]

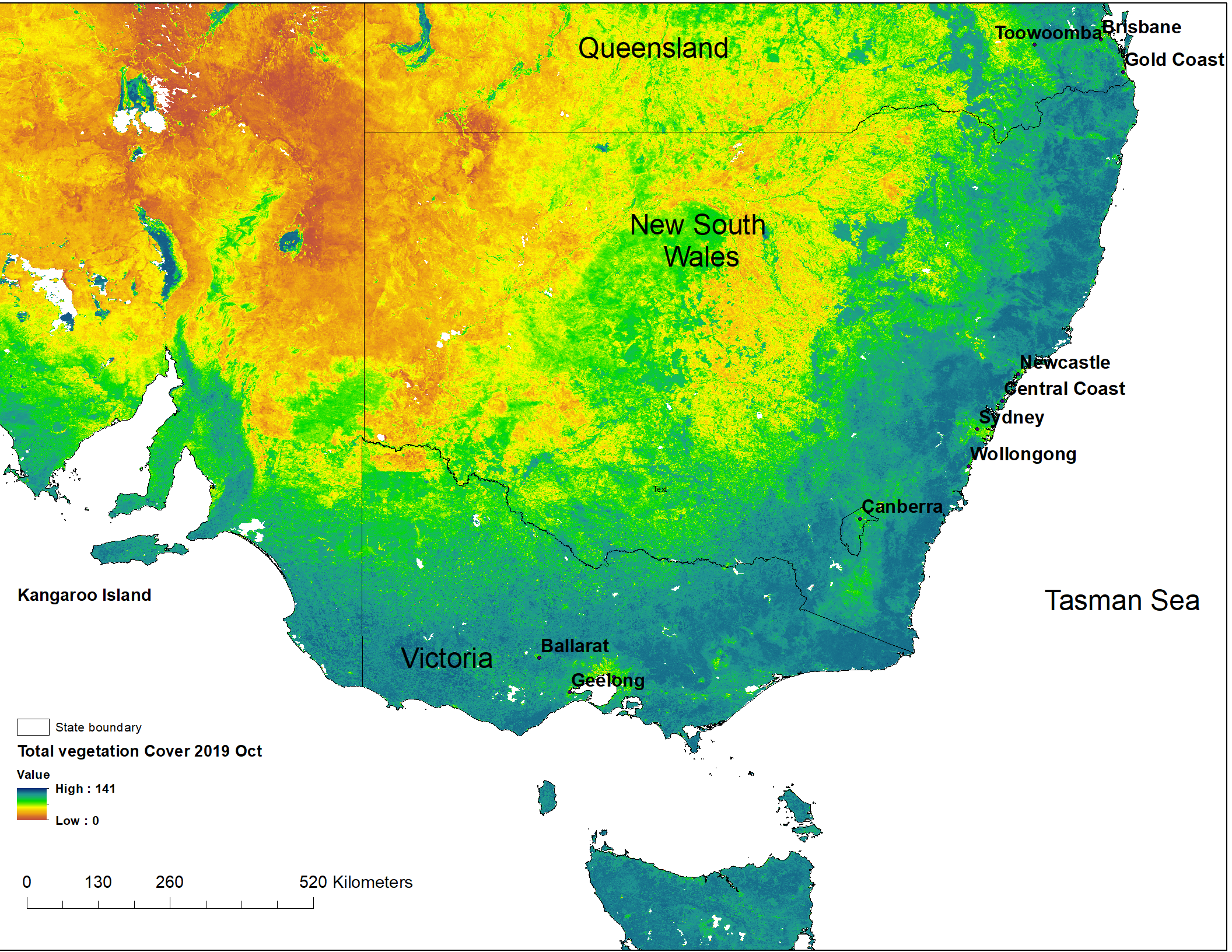

Supplement: Supplementary file 1 [file ijerph-18-03538-s001.zip › vegetation_cover_2019-oct-seaus2.tif]
